# Lattice Dynamics in the NASICON NaZr_2_(PO_4_)_3_ Solid Electrolyte from Temperature-Dependent Neutron Diffraction, NMR, and Ab Initio Computational Studies

**DOI:** 10.1021/acs.chemmater.2c00212

**Published:** 2022-04-28

**Authors:** Emily E. Morgan, Hayden A. Evans, Kartik Pilar, Craig M. Brown, Raphaële J. Clément, Ryo Maezono, Ram Seshadri, Bartomeu Monserrat, Anthony K. Cheetham

**Affiliations:** † Materials Department and Materials Research Laboratory, University of California, Santa Barbara, California 93106, United States; ‡ NIST Center for Neutron Research, National Institute of Standards and Technology, Gaithersburg, Maryland 20878, United States; ¶ School of Information Science, Japan Advanced Institute of Science and Techology, Asahidai 1-1, Nomi, Ishikawa 923-1292, Japan; § Department of Chemistry and Biochemistry, University of California, Santa Barbara, California 93106, United States; ∥ Department of Materials Science and Metallurgy, University of Cambridge, 27 Charles Babbage Road, Cambridge CB3 0FS, United Kingdom; ⊥ Cavendish Laboratory, University of Cambridge, J. J. Thomson Avenue, Cambridge CB3 0HE, United Kingdom; # Department of Materials Science and Engineering, National University of Singapore, Singapore 117575, Singapore

## Abstract

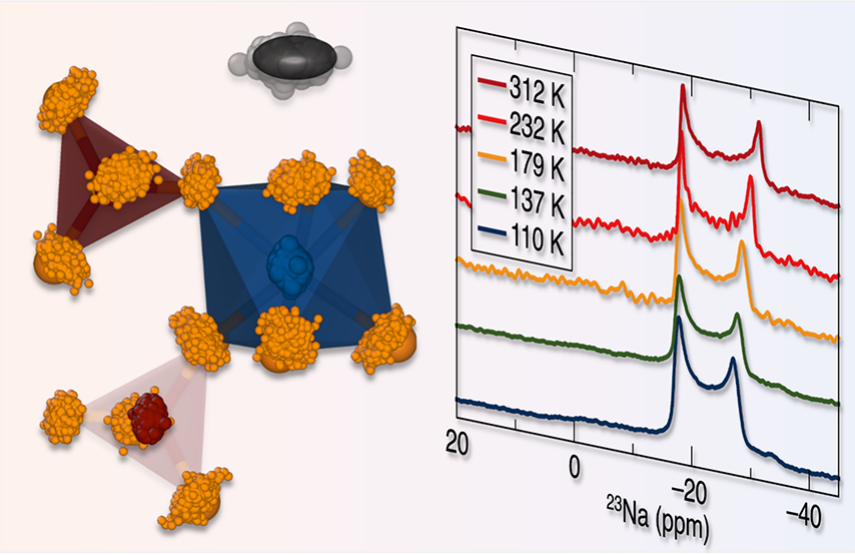

Natrium super ionic conductor (NASICON) compounds form a rich and highly chemically tunable family of crystalline materials that are of widespread interest because they include exemplars with high ionic conductivity, low thermal expansion, and redox tunability. This makes them suitable candidates for applications ranging from solid-state batteries to nuclear waste storage materials. The key to an understanding of these properties, including the origins of effective cation transport and low, anisotropic (and sometimes negative) thermal expansion, lies in the lattice dynamics associated with specific details of the crystal structure. Here we closely examine the prototypical NASICON compound, NaZr_2_(PO_4_)_3_, and obtain detailed insights into such behavior via variable-temperature neutron diffraction and ^23^Na and ^31^P solid-state NMR studies, coupled with comprehensive density functional theory-based calculations of NMR parameters. Temperature-dependent NMR studies yield some surprising trends in the chemical shifts and the quadrupolar coupling constants that are not captured by computation unless the underlying vibrational modes of the crystal are explicitly taken into account. Furthermore, the trajectories of the sodium, zirconium, and oxygen atoms in our dynamical simulations show good qualitative agreement with the anisotropic thermal parameters obtained at higher temperatures by neutron diffraction. The work presented here widens the utility of NMR crystallography to include thermal effects as a unique probe of interesting lattice dynamics in functional materials.

## Introduction

**Figure 1:**
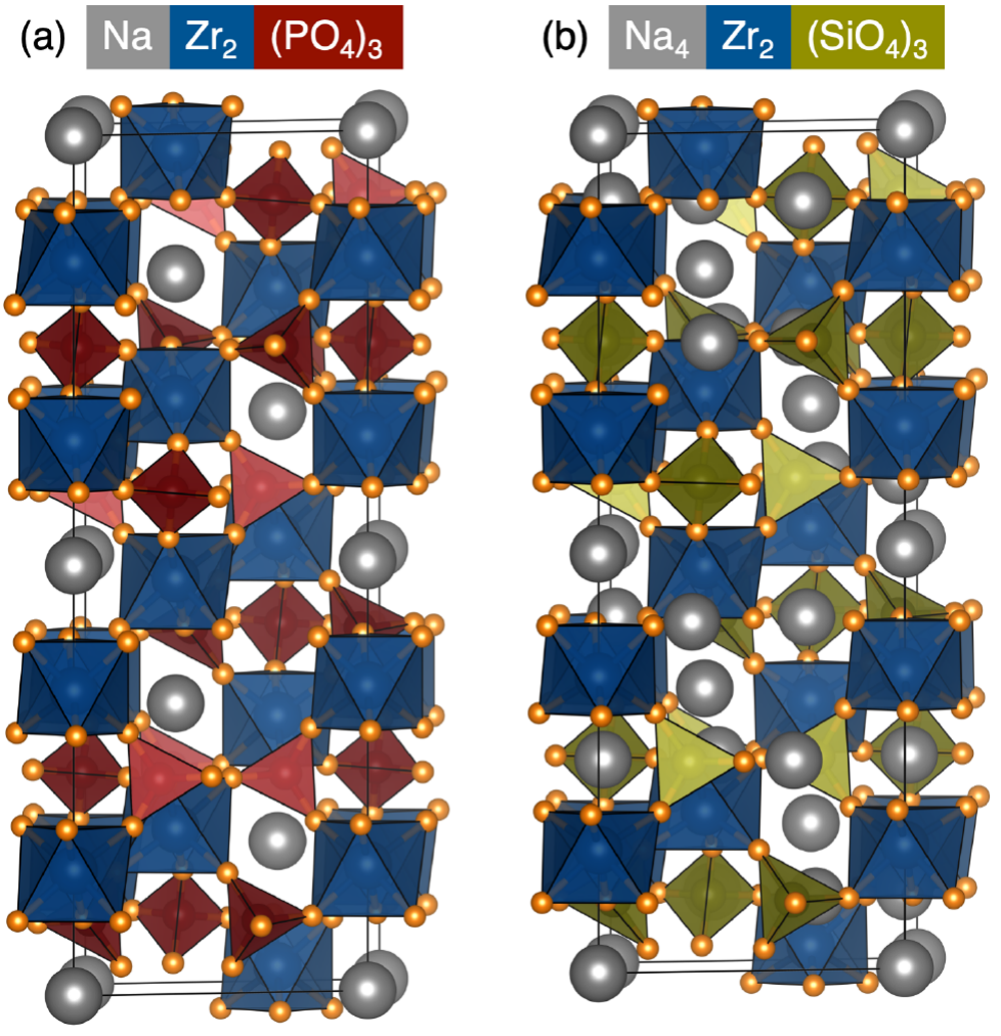
Two end members of the Na_1+*x*_Zr_2_P_3–*x*_Si_*x*_O_12_ family of compounds: (a) NaZr_2_(PO_4_)_3_.^3^ (b) Na_4_Zr_2_(SiO_4_)_3_.^12^

The crystal structure of NaZr_2_(PO_4_)_3_ was first reported in 1968^1^ and attracted renewed interest beginning in 1976, as NaZr_2_(PO_4_)_3_ displayed exceptional ionic conductivity^2,3^ and low coefficients of thermal expansion.^4^ These NASICONs, from natrium super ionic conductors, have been established as promising materials for a variety of applications, most notably in the field of energy storage.^5−7^ The original family of NASICON materials have the general formulaNa_1+*x*_Zr_2_P_3–*x*_Si_*x*_O_12_, where the two end-member crystal structures are shown in Figure 1. These structures are both composed of a network of corner-sharing MO_6_ octahedra and XO_4_ tetrahedra, in which the combination of two MO_6_ octahedra joined by three XO_4_ tetrahedra forms the characteristic “lantern” subunits. The compounds adopt the spacegroup *R*3̅*c* (or lower symmetry subgroups thereof), with the lantern subunits stacked parallel to the [001] direction in the hexagonal setting. These subunits form an open structure such that up to four Na^+^ cations can be accommodated in the interstitial spaces. The stoichiometry of the compound can be used to tune the mobility of these ions, and many substitutions are possible on the atomic positions, following the overall formula A_*x*_MM^′^(XO_4_)_3_. In turn, these substitutions enable the precise tuning of properties such as ionic and electronic conductivity and thermal expansion, making these ideal materials for solid-state electrolytes, electrodes,^5−7^ and nuclear waste storage.^8^ Many compositions of the NASICON structure have been synthesized and characterized since its discovery, and computational studies continue to highlight new phases for use in these applications,^9−11^ illustrating the thriving interest in this class of compounds.

The most well-known application for NASICON compounds is for use in batteries, where the versatility of the crystal structure makes it suitable as both an electrolyte and electrode material for lithium- and sodium-ion batteries. In addition to the zirconium-based family of materials, some other common compositions include NASICONs containing iron, vanadium, and titanium.^5−7^ For example, Na_3_MnZr(PO_4_)_3_ has been recently reported as a potential high-voltage cathode material for sodium-ion batteries,^13^ while Na_3_Ti_2_(PO_4_)_3_ has been proposed for low-voltage anode applications.^14^ Similarly, LiZr_2_(PO_4_)_3_^15^ and Li_1.3_Al_0.3_Ti_1.7_(PO_4_)_3_^16^ are promising electrolytes for all-solid-state lithium batteries. The flexibility of the NASICON structure has also allowed for the development of battery materials containing mobile divalent cations, such as calcium^17^ or zinc.^18^ Finally, in some recent studies, NASICON compounds have also been examined for applications in unconventional battery architectures, such as seawater^19^ or redox-flow batteries.^20^

In addition to their tunable ionic and electronic conductivity, one of the key characteristics that makes NASICON compounds desirable for energy storage is their low thermal expansion. In NASICON-type materials, systematic lattice substitutions, diffraction, and dilatometry experiments have been used to construct a model for the thermal expansion behavior.^4,21−27^ These studies have shown that when temperature is increased, the Na^+^ sites become larger and the Na–O bond distances increase, driving an expansion along the *c* axis. To minimize distortions of the ZrO_6_ octahedra and PO_4_ tetrahedra, the polyhedra undergo coordinated rotations with respect to one another, resulting in a decrease in the *a* lattice parameter. Depending on the specific composition of the NASICON material, the relative magnitudes of the changes in the *a* and *c* lattice parameters can vary, causing the thermal expansion to be negative, positive, or nearly zero.^22^

While static models have been successful in explaining the low thermal expansion of NASICON materials, understanding local dynamics in the crystal structure is essential to explaining the mechanisms of ion conduction. As discussed in a recent review,^28^ phonon mode calculations may provide a promising route for the discovery of new solid-state electrolytes. For example, it has been shown that there is good correlation between the frequency or amplitude of certain phonon modes and activation energies for diffusion or migration barriers in systems such as cubic metals,^29^ metal halides,^30^ and Ruddlesden–Popper phases.^31^ While these parameters are computationally accessible, they can be more difficult to observe directly in experiment, generally requiring the use of inelastic neutron or X-ray scattering.^28^ Lattice dynamics have successfully been used to understand the diffusion of Na^+^ ions in Sc-doped NASICON materials.^32^ In these materials it was shown that higher concentrations of Sc^3+^ increase the activation energy for ion diffusion, resulting in a lower ionic conductivity. Furthermore, while long-range Na^+^ diffusion occurs via three-dimensional pathways, diffraction data and NMR relaxation measurements show that local motion of the Na^+^ ions is two-dimensional, which is important in modeling the hopping of the Na^+^ ions between sites. This type of study illustrates the value of connecting local structural dynamics with bulk material properties such as conductivity.

Both the exciting ionic conductivity and low thermal expansion of NASICON materials, as well as past work on their structure–property relationships, have motivated us to examine the structure of the parent compound, NaZr_2_(PO_4_)_3_, more closely, using a combination of powder neutron diffraction, solid-state NMR, and DFT calculations. These techniques can be used to gain insight into how local dynamics describe ionic conductivity and thermal expansion in this class of materials. Overall, the results of the neutron diffraction study are consistent with previous models of thermal expansion in NaZr_2_(PO_4_)_3_, with the *a* lattice parameter decreasing as the temperature is increased from 25 to 400 K, while the *c* lattice parameter increases over this same temperature range. Additionally, the atomic displacement parameters (ADPs) for each site were determined for each temperature, and the values were compared with the results of DFT phonon mode calculations. Variable temperature ^23^Na and ^31^P NMR experiments from 100 to 300 K revealed a systematic increase in the ^31^P chemical shift and ^23^Na *C*_Q_ parameter (which describes the asymmetry of the charge distribution around the ^23^Na site) that reflects changes in the dynamics of this material over this temperature range. In light of these results, static DFT calculations were performed to predict NMR parameters for the neutron structures obtained at various temperatures. These calculations, which did not account for thermal motion, did not reproduce the experimental trends, providing evidence that the trend in the NMR parameters is not exclusively due to the thermal expansion of the lattice but instead reflects the effects of vibrational motion on local atomic environments. Additional calculations were therefore performed which incorporated both the thermal expansion of the lattice and phonon modes into the prediction of NMR parameters. In this case, the experimental trends were reproduced qualitatively.

## Experimental Section

### Synthesis of NaZr_2_(PO_4_)_3_

Solid-state synthesis of NaZr_2_(PO_4_)_3_ was based on the original synthesis reported by Hong.^3^ Thus, 2.34 mmol (0.2484 g) of Na_2_CO_3_, 8.16 mmol (1.0056 g) of ZrO_2_, and 12.23 mmol (1.6156 g) of (NH_4_)_2_HPO_4_ were combined and ground in a mortar and pestle for 20 min. The powder was placed in an alumina crucible and heated to 1273 K (1000 °C) at a rate of 4 K per min. The temperature was held at 1273 K (1000 °C) for 16 h before cooling to room temperature.

### Powder Neutron Diffraction and Rietveld Refinements

Measurements were performed on 1.7 g of NaZr_2_(PO_4_)_3_ at the National Institute of Standards and Technology Center for Neutron Research (NCNR). Data were collected at the high-resolution neutron powder diffractometer, BT-1, utilizing a Cu(311) monochromator with an in-pile 60° collimator, corresponding to a neutron wavelength of 1.540 Å. The sample was loaded into a vanadium sample can in a He-environment glovebox and sealed with an indium O-ring onto a copper heating block. After mounting the sample onto a bottom-loaded closed cycle refrigerator (CCR), the sample was cooled and then measured upon heating upward from 25 to 400 K. Data were collected for at least 4 h for each temperature point.

The powder neutron diffraction data were analyzed using the GSAS software suite.^33^ Initial Le Bail refinements were conducted to determine lattice parameters and peak shapes.^34^ Background, zero-point error, atomic positions, and isotropic ADPs were refined for all Rietveld refinements in space group *R*3̅*c* (167). For the 100 K through 400 K data sets, anisotropic ADPs were refined for the sodium site. Additionally, for the 325 and 400 K data sets, anisotropic ADPs were also refined for the oxygen and zirconium sites. Isotropic ADPs were used for all other temperatures and sites not specified here.

### NMR Measurements

NMR spectra were acquired in the temperature range of 100 to 300 K using a Bruker 400 MHz (9.4 T) Ascend DNP-NMR spectrometer equipped with a 3.2 mm MAS DNP-NMR triple resonance broadband X/Y/H magic angle spinning (MAS) probe while spinning at a rate of 8 kHz. At each temperature point, the sample temperature was calibrated using the T_1_ of ^79^Br in KBr.^35^ For the ^23^Na measurements, the probe was tuned to 105.9 MHz and a zg experiment with a π/2 pulse of 6 μs at 10 W was used to selectively excite the central transition. The recycle delay was set to 40 s. ^23^Na spectra are reported with respect to the chemical shift of a 1.0 M solution of NaCl in water set to 0 ppm. For ^31^P spectra, the probe was tuned to 162.0 MHz and a zg experiment with a π/6 pulse of 1 μs at 160 W was used. The recycle delay was set to 200 s. All ^31^P spectra are reported with respect to the chemical shift of solid triphenylphosphine set to −9 ppm. Spectra were simulated using the SOLA module in the TopSpin software package.

### DFT Calculations

For both the standard and finite temperature NMR calculations, chemical shifts and quadrupolar coupling constants were determined using density functional theory,^36,37^ the PBE functional,^38^ and the GIPAW formalism^39,40^ as implemented in the CASTEP package,^41^ using an energy cutoff of 800 eV, a **k**-point grid spacing of 2π × 0.025 Å^–1^,^42^ and ultrasoft pseudopotentials.^43^ For structure relaxations, the cell parameters were fixed to the experimentally measured values at each considered temperature, and the internal coordinates of the atoms were relaxed until all forces were smaller than 10^–4^ eV/Å.

Phonon calculations were performed using the finite displacement method^44^ in conjunction with nondiagonal supercells.^45^ The calculations revealed the presence of imaginary modes at a few **q**-points located at the Brillouin zone boundary. A mapping of the energy as a function of the amplitude of the imaginary modes showed a shallow quartic double-well potential. The self-consistent harmonic approximation was then applied to the individual imaginary modes,^46,47^ and as a result the imaginary frequencies became real at all finite temperatures considered in this work. These results indicate that anharmonic lattice dynamics are necessary to stabilize the structure.

In the finite temperature calculations, the calculated phonon modes were then used to stochastically generate atomic positions distributed according to the vibrational density at the corresponding temperature,^48^ and accelerated using thermal lines.^49,50^ The finite temperature NMR parameters were calculated with appropriate averages over configurations. The atomic positions generated in this process were visualized using the OVITO software package.^51^ The 2D projections of these distributions are available in the Supporting Information. For the finite temperature calculations, the chemical shielding was converted to chemical shift using the formula: δ = σ^ref^ – σ, where σ^ref^ was chosen such that the calculated shift for the 100 K structure would correspond to that observed experimentally near 100 K. For the standard DFT calculations, to convert from the calculated chemical shielding to an experimental chemical shift, calibration curves were constructed by plotting the calculated shielding for several relevant phosphates and sodium-containing compounds against their experimental chemical shift values from the literature.^52−57^ More information regarding these calibration curves, as well as alternative methods for rescaling the chemical shieldings, can be found in the Supporting Information.

## Results and Discussion

**Figure 2:**
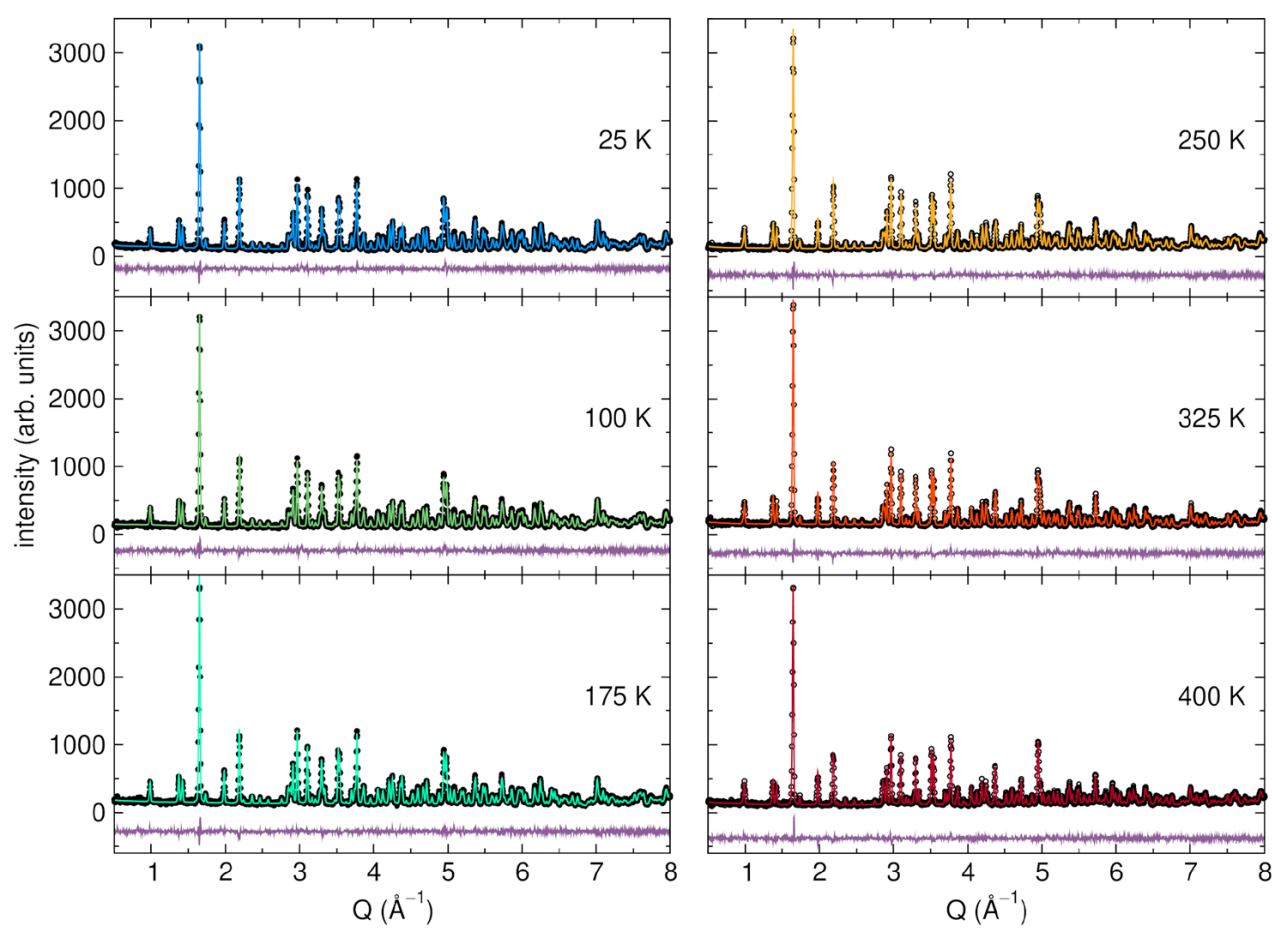
Neutron diffraction data (BT-1, NCNR, wavelength = 1.540 Å) as a function of *Q* (defined as 2π/λ) and Rietveld refinements at temperatures from 25 to 400 K. Black circles represent the experimental data while colored lines represent the Rietveld refinement fits. The purple line below shows the difference between experimental data and Rietveld refinement fits. The Rietveld refinement statistics for each fit are 25 K: *R*_wp_ = 7.18%, *R*_p_ = 5.82%; 100 K: *R*_wp_ = 6.91%, *R*_p_ = 5.63%; 175 K: *R*_wp_ = 6.76%, *R*_p_ = 5.56%; 250 K: *R*_wp_ = 6.99%, *R*_p_ = 5.77%; 325 K: *R*_wp_ = 6.66%, *R*_p_ = 5.42%; 400 K: *R*_wp_ = 6.80%, *R*_p_ = 5.56%;

As shown in Figure 2, powder neutron diffraction data were collected for NaZr_2_(PO_4_)_3_ between 25 and 400 K. Rietveld refinements were performed for each data set to determine the lattice parameters, atomic positions, and atomic displacement parameters for the structure as a function of temperature. The calculated differences between the experimental data and the Rietveld refinements illustrate the high quality of both the data and the fits.

**Figure 3:**
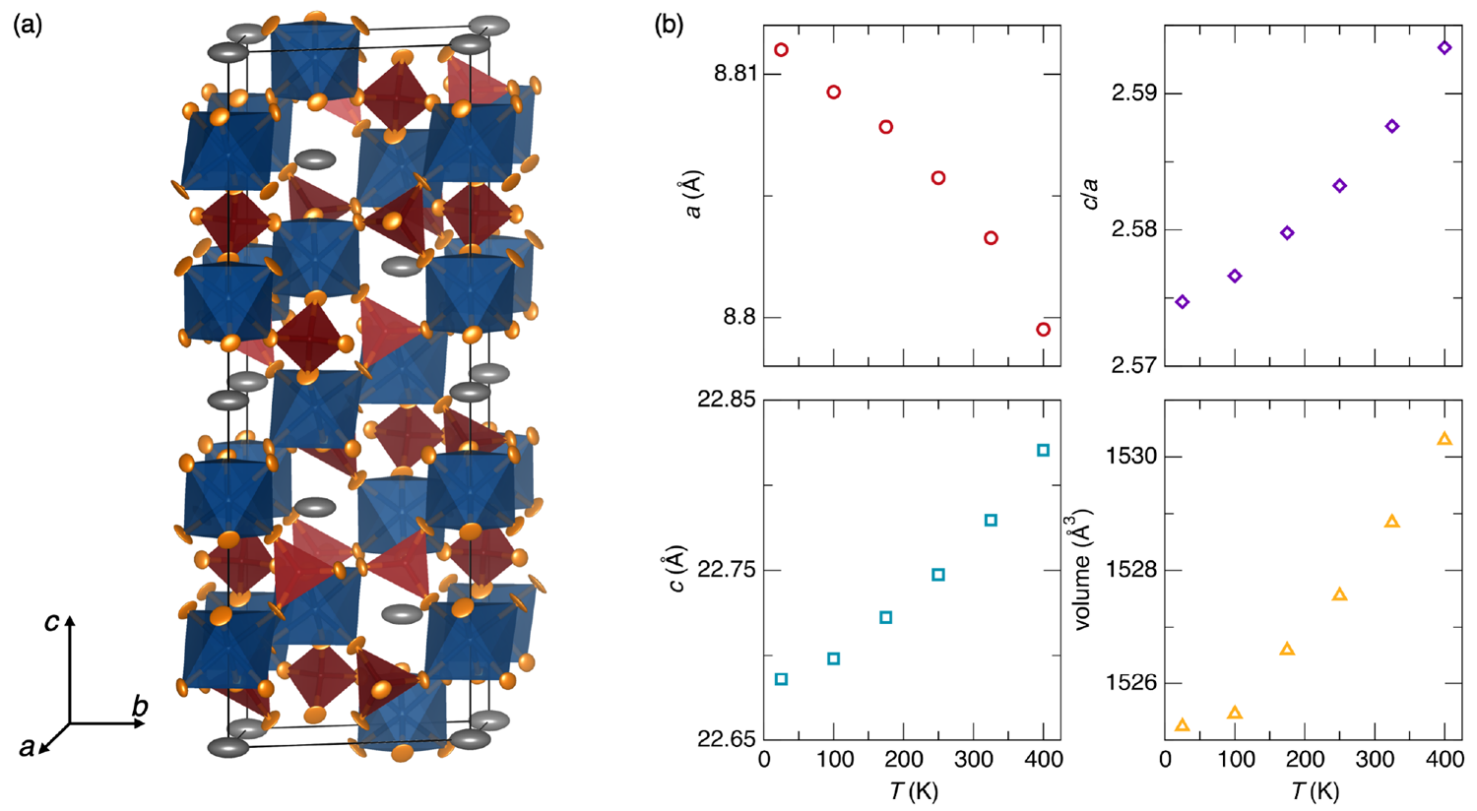
(a) Representative crystal structure for NaZr_2_(PO_4_)_3_ at 325 K with atomic displacement parameters shown at 95%. Anisotropic parameters were refined for the sodium, oxygen, and zirconium sites, while isotropic parameters were used for the phosphorus site. (b) Lattice parameters and cell volumes for NaZr_2_(PO_4_)_3_ at temperatures from 25 to 400 K. Error bars representing one standard deviation are commensurate with symbol size.

The structures determined from the Rietveld refinements are in good agreement with previous studies of the NASICON family. The structure is composed of chains of corner-sharing ZrO_6_ octahedra and PO_4_ tetrahedra which form an open network, with Na^+^ localized in octahedral sites at (0, 0, 0), as shown in Figure 3(a). In Si-substituted members of the NASICON family, the Na^+^ ions occupy a second site in the structure to balance the added charge from the Si^4+^; however, in NaZr_2_(PO_4_)_3_ there was no evidence for Na^+^ occupation of this second site, even at high temperatures. Additionally, the thermal expansion behavior of this material is consistent with earlier reports.^4^ Upon heating from 25 to 400 K, the *a* lattice parameter decreases from 8.81101(11) Å to 8.79945(18) Å, while the *c* lattice parameter increases from 22.6859(5) Å to 22.8199(7) Å. As a result, the volume of the unit cell increases by only 0.33% over this temperature range, as illustrated in Figure 3(b).

**Figure 4:**
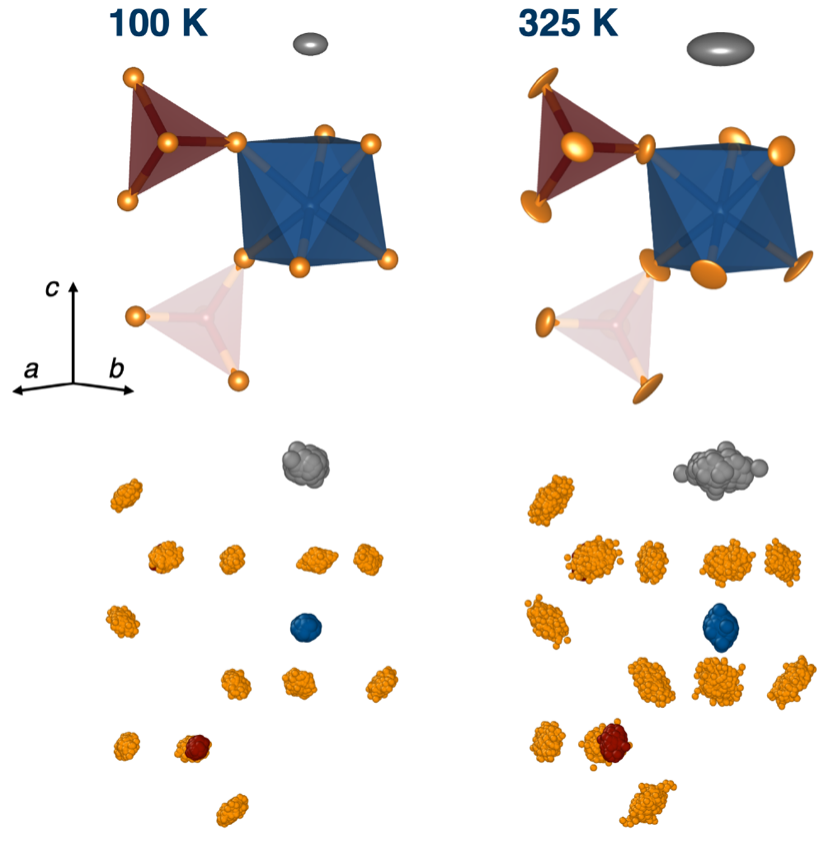
Representative sections of the crystal structure for NaZr_2_(PO_4_)_3_ at 100 and 325 K. The top portion of the figure shows the structure determined from the neutron refinements, with atomic displacement parameters shown at 95%. In the 100 K structure, anisotropic ADPs were refined only for the sodium site, and isotropic ADPs were used for all other sites. At 325 K, anisotropic parameters were refined for the sodium, oxygen, and zirconium sites, while isotropic parameters were refined for phosphorus. The bottom portion of the figure shows images composed of 1000 structures which were stochastically generated following the atomic distribution as determined by the DFT phonon calculations. In these structures each individual atom is shown at 10% of its default radius to clearly visualize the shape of each cluster.

Figure 4 shows representative fragments of the structure at 100 and 325 K, where these temperatures were selected because this is the range of temperatures that was later accessible in the NMR studies. These fragments illustrate several important features of the structure and its thermal expansion behavior. First, the sodium sites expand with increasing temperature, with the sodium–oxygen bond distance increasing from 2.5345(13) Å at 100 K to 2.5643(18) Å at 325 K (+0.0298 Å, +1.18%). Furthermore, the ADPs indicate that as the temperature increases, the Na atom spends a greater amount of time away from the high-symmetry (0, 0, 0) position. The *U* value for sodium increases from 0.0133 Å^2^ at 100 K to 0.0450 Å^2^ at 325 K. Furthermore, the sodium site becomes more anisotropic, with the ratio *U*_11_/*U*_33_ increasing from 2.31 to 4.00. In contrast, the phosphorus and zirconium sites show only small changes. At 100 K, the two unique P–O bond distances in the structure are 1.5262(18) Å and 1.5289(16) Å, while at 325 K, the bond distances are 1.526(3) Å and 1.525(3) Å (changes of −0.0002 Å, −0.01% and −0.0039 Å, −0.26%, respectively). Similarly, the zirconium–oxygen bond distances remain constant or decrease slightly with temperature. At 100 K, the two unique Zr–O bond distances are 2.040(2) Å and 2.0937(18) Å, and at 325 K, they are 2.041(3) Å and 2.085(3) Å (changes of +0.001 Å, +0.05% and −0.0087 Å, −0.4%, respectively). In both the PO_4_ and ZrO_6_ polyhedra, the bond angles change only slightly over this temperature range (see the Supporting Information for complete bond length and angle information). This confirms earlier assumptions in the literature that, based on average bond distances, the PO_4_ tetrahedra and ZrO_6_ octahedra undergo only very small distortions over the range of temperatures studied.

To further investigate the changes in atomic motion and local distortions with temperature, the DFT phonon mode calculations were used as a starting point to generate 1000 structures at each temperature in which the atomic positions are distributed according to the harmonic density. The resulting structures are overlaid to visualize the thermal motion, as shown in Figure 4 (an alternative view of the structures is available in the Supporting Information).

**Figure 5:**
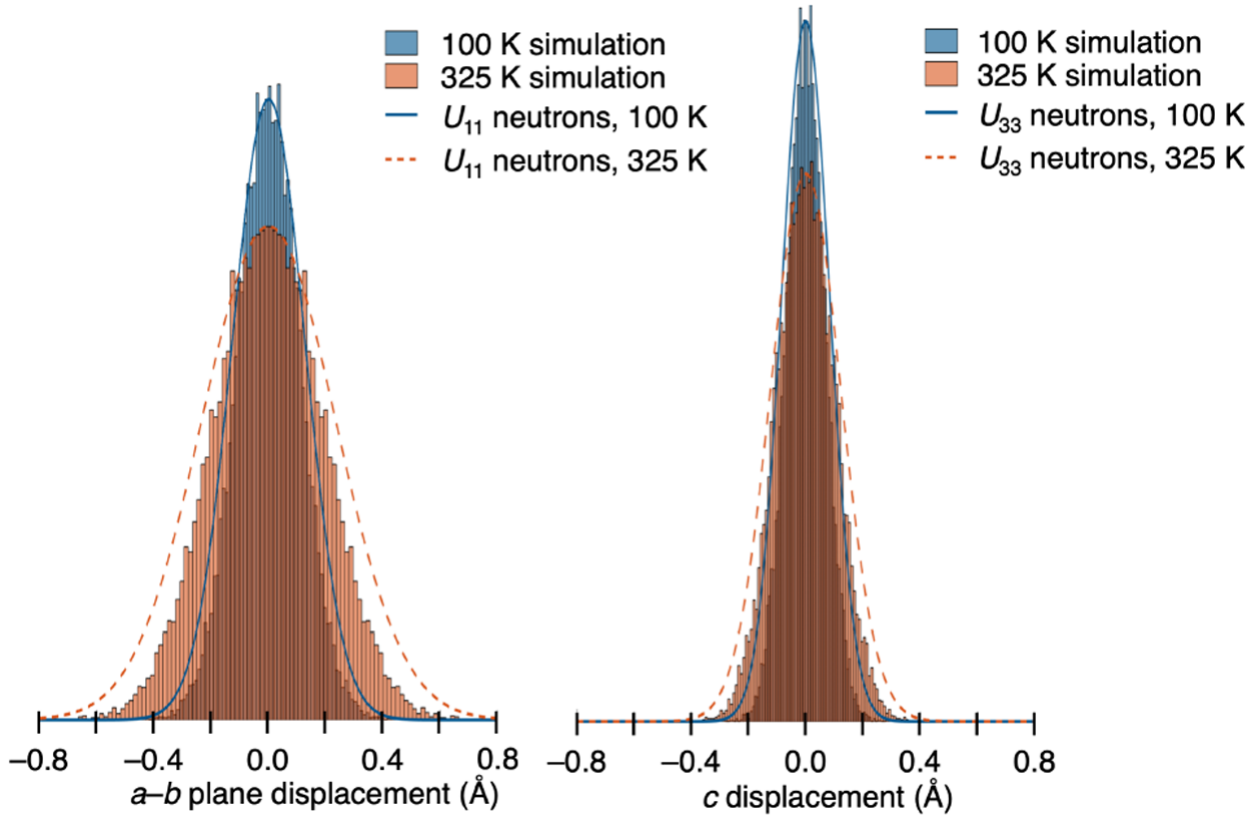
Histograms depicting the distribution of displacements away from the equilibrium position for the sodium site in the *a–b* plane and *c* direction at 100 and 325 K. The overlaid curves represent the expected distribution of positions based on the *U*_11_ and *U*_33_ values from the neutron refinements at 100 and 325 K.

This figure illustrates the good agreement between the neutron and computational data, particularly for the sodium site, where the configurations generated from DFT also show a significant increase in the size and anisotropy of the sodium site. For this site, it was also possible to perform a more detailed quantitative comparison between the *U* values from the neutron structures and the displacements of the atoms in the computational results, as shown in Figure 5. The histograms clearly show the similarity between the computational distribution of sodium positions and the distribution that would be expected based on the *U*_11_ and *U*_33_ values determined from the neutron refinements. There are some minor discrepancies between the two, with the computational mean squared displacements tending to be smaller than the corresponding experimental *U* values. The best agreement between the two is found at lower temperatures, indicating that anharmonic thermal motion becomes important at the higher temperature range (see the Supporting Information for a full list of *U* values and comparison with mean squared displacements from computational data).

The computational positions for the phosphorus and zirconium sites agree qualitatively with the neutron data, although the mean squared displacements from the computational data are significantly larger than the *U*_iso_ values obtained from the neutron refinements. In both data sets, the phosphorus and zirconium sites have smaller mean squared displacements which increase a small amount with temperature in comparison with the sodium and oxygen sites. Additionally, in the case of the zirconium site which displays some anisotropy at higher temperatures, both the neutron ADPs and the computational mean squared displacements show a greater degree of motion along the *c* axis than in the *a–b* plane. Finally, computational and neutron results for the oxygen positions are qualitatively similar, although comparison with the neutron ADPs is slightly complicated by the fact that oxygen anisotropic ADPs could only be refined for the 325 and 400 K data sets. For the lower temperature data sets, the anisotropic ADPs became physically unrealistic, and therefore isotropic ADPs were determined to be more appropriate. In the computational data, the average mean-squared displacement from the equilibrium positions increases by a factor of 3 between 100 and 325 K, and the distribution becomes more anisotropic as the temperature increases. In the neutron refinements, the *U* values are much smaller than the mean-squared displacements in the computational data, but the *U*_iso_ values do increase by a factor of 2 between 100 and 325 K. Additionally, the orientations of the neutron ADPs in the 325 K data set appear to be qualitatively similar to the distributions predicted by computation.

**Figure 6:**
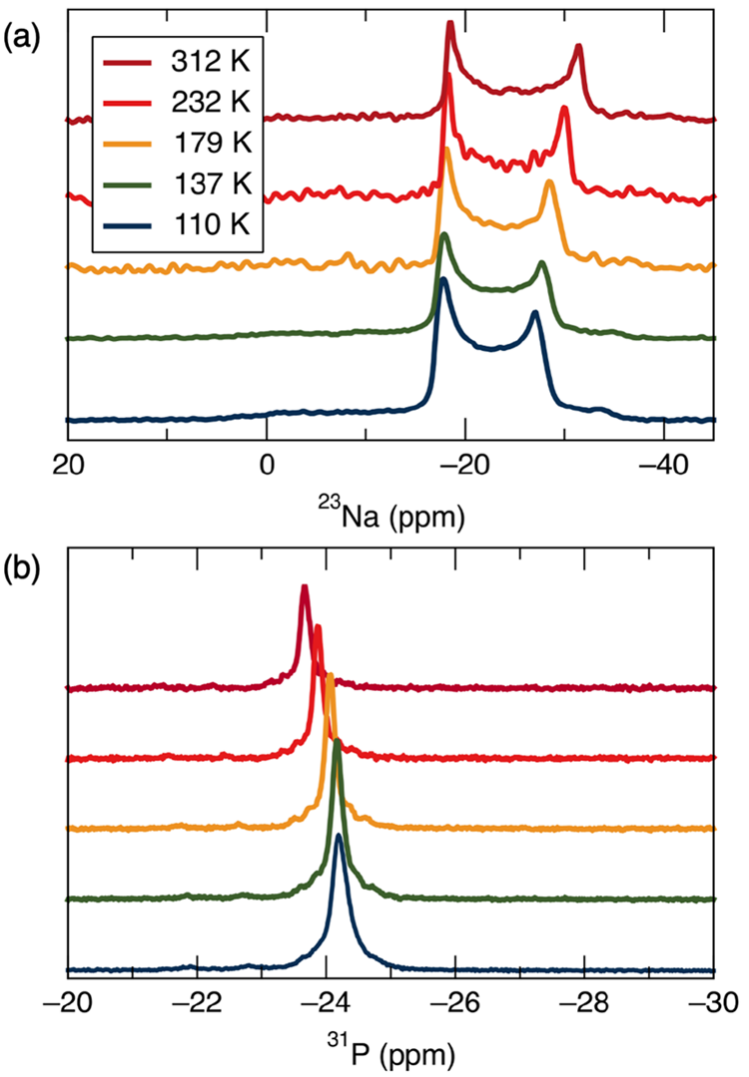
(a) Variable temperature ^23^Na spectra. (b) Variable temperature ^31^P spectra.

Variable-temperature NMR experiments provide an effective way to probe the influence of temperature on local structure and dynamics in a material. The spectra for ^31^P and ^23^Na taken between approximately 100 and 300 K are shown in Figure 6. These spectra display several interesting trends over the range of temperatures measured. First, because ^23^Na has a nuclear spin of *I* = 3/2, it is a quadrupolar nucleus and therefore is described in terms of both its chemical shift tensor and electric field gradient tensor. Simulations of the spectra indicate that the isotropic chemical shift increases slightly from −14.23 ppm to −14.18 ppm between 100 and 300 K. For quadrupolar nuclei, the observed chemical shift is the sum of the isotropic chemical shift and the second-order quadrupolar shift; however, for simplicity, we focus on the isotropic shift here. Additionally, the quadrupolar coupling constant, *C*_Q_, which describes the width of the line shape, increases from 1.96 to 2.24 MHz. Initially, the trends in NMR parameters were compared with the structures from the neutron diffraction data and trends in NMR parameters from previous studies of similar materials. While some of the trends could be rationalized using this strategy, the cause of others remained unclear. For example, given the significant increase in the Na–O bond distances between 100 and 300 K, a larger change in the chemical shift was anticipated. Additionally, previous studies of the NMR parameters of sodium silicates found that the chemical shift decreases with increasing Na–O bond length,^53^ so it was unexpected that the chemical shift increased slightly instead. The *C*_Q_ parameter reflects the asymmetry of the charge distribution around the ^23^Na site, where lower values of the coupling constant indicate a more symmetrical site. The increase in *C*_Q_ is consistent with the results of the neutron diffraction, which show the Na vibrations becoming more anisotropic at higher temperatures (Figure 4).

In the ^31^P spectra there is a significant increase in the isotropic chemical shift, from −24.19 ppm to −23.66 ppm. In this case, the chemical shift was not expected to change significantly with temperature, as neutron diffraction data indicated that the PO_4_ tetrahedra are fairly rigid. There have been several investigations of strategies for interpreting and predicting the ^31^P chemical shifts in phosphates; however, they cannot completely explain the trends observed for NaZr_2_(PO_4_)_3_.^54,58−61^ For example, Cheetham et al.^52^ found that weaker P–O bonds are associated with larger chemical shifts, but given the fact that there is no strong trend in the average bond distances, it is unlikely that this is the correct explanation for our NaZr_2_(PO_4_)_3_ results.

Given that the trends in the NMR data could not be completely explained by the neutron diffraction structures, DFT calculations were performed to predict the NMR parameters at each temperature. Three different strategies for calculating the NMR parameters were attempted. For model 1, the NMR parameters were directly calculated from the neutron structures, which should capture any trends in the chemical shift or *C*_Q_ values due to the thermal expansion of the lattice. In model 2, a DFT structural relaxation was performed for each neutron structure where the lattice parameters were fixed at each temperature and the atomic positions allowed to relax, followed by the NMR calculation. This technique was chosen because several studies on performing DFT calculations of NMR parameters have shown that the agreement between theory and experiment can be improved significantly by using relaxed structures.^58,62^ Finally, in model 3, the NMR parameters were determined by averaging over the stochastic positions generated by the previously discussed phonon mode calculations, to incorporate the effects of both the thermal expansion of the lattice and atomic vibrations.

**Figure 7:**
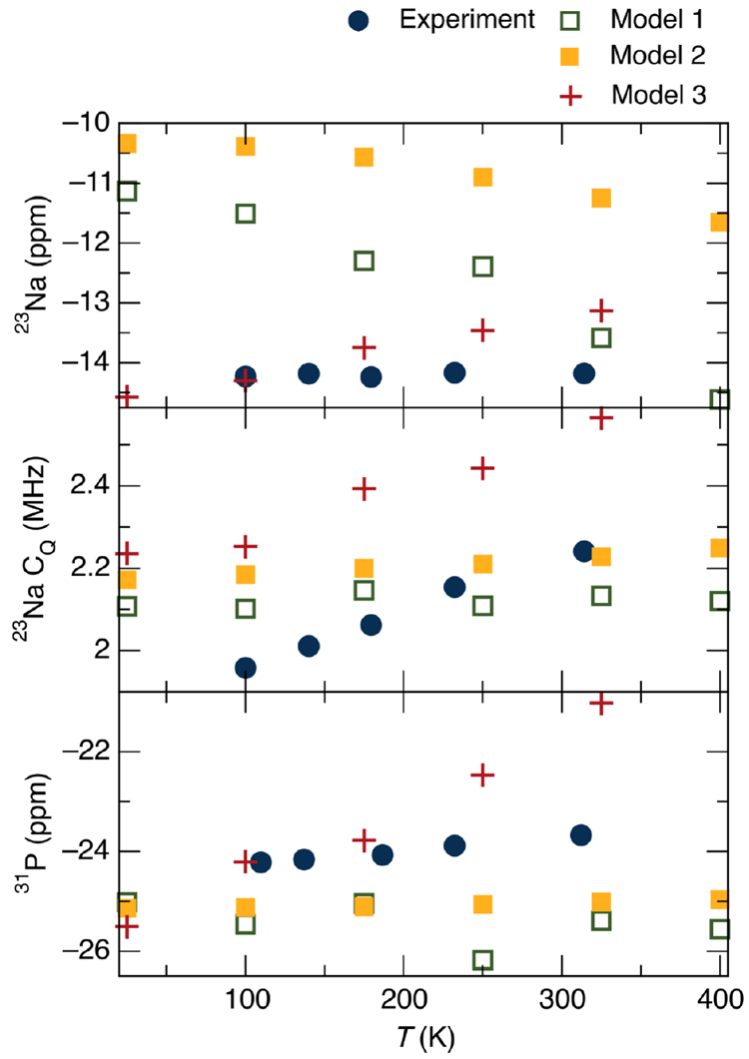
Comparison of experimental NMR parameters with those predicted by DFT at different levels of theory.

The results of these three sets of calculations and comparison with the experimental results are shown in Figure 7. Beginning with model 1, it is clear that these results do not correspond well with what is observed experimentally, but they are similar to what might be predicted exclusively based on the thermal expansion of the lattice. For example, the ^23^Na chemical shift decreases significantly with temperature and increasing Na–O bond length, which is similar to what has been observed experimentally in other systems. Additionally, there is not a strong trend in the *C*_Q_ parameter, which makes sense because this type of DFT calculation cannot incorporate the information communicated by the atomic displacement parameters. The ^31^P chemical shift does not change with temperature, which is also what could be predicted from the neutron structures. These results further illustrate the point that the observed NMR trends do not simply originate from the thermal expansion of the lattice. We note here that trends in calculated chemical shifts can depend somewhat on the method used to convert between chemical shielding and chemical shift. In this work we use the calibration curves discussed in Experimental Section and have confirmed that the general trends observed in models 1 and 2 do not change significantly when other rescaling methods are used. More detail can be found in the Supporting Information.

Considering model 2, the trends seem to be closer to those observed experimentally, but there remains a significant difference between the two. The relaxed results show a very small increase in both the ^23^Na *C*_Q_ and the ^31^P chemical shift. These trends qualitatively match experimental results, but in both cases the magnitude of the increase is much less than that found in experiment. In the case of the sodium, the Na–O bonds in the relaxed structures are shorter than in the neutron structures and do not increase as much with temperature (relaxed distances are 2.4879 and 2.4965 Å at 100 and 325 K). This is consistent with the fact that the ^23^Na chemical shift shows a smaller decrease than is found in model 1 but is still far from matching the experimental results. Similarly, the P–O distances distances were both 1.5408 Å at 100 K and 1.5410 and 1.5409 Å at 325 K, which may account for the small increase in ^31^P chemical shift. Overall, however, due to the subtle changes in the structure upon relaxation, it is difficult to conclusively determine which aspects of the structural relaxation contribute to the differences in the NMR parameters between models 1 and 2. This is similar to the findings of other DFT studies of phosphates, where structural relaxations significantly improve the correlation between experimental and predicted chemical shifts, despite the fact that the NMR chemical shifts are measured at ambient temperature while these DFT calculations do not explicitly account for thermal effects associated with atomic vibrations.^58^

While NMR calculations from relaxed structures show slightly better performance than those from diffraction-based structures, these calculations do not provide a satisfactory explanation for the experimental NMR trends. Therefore, in model 3, NMR parameters were calculated as thermal averages over the atomic vibrational motion for each of the relaxed neutron structures, as described in Experimental Section. As shown in Figure 7, the results of model 3, which explicitly account for thermal motion in the structures, show better qualitative agreement with our experimental trends. For the chemical shifts, the magnitudes of the calculated shifts are less meaningful in some ways because the value of σ^ref^ was chosen somewhat arbitrarily, as described in Experimental Section. This differs from models 1 and 2, where a calibration curve was used to determine this value. However, more significant is the fact that the finite temperature calculations predict an increase in both chemical shifts, as well as the *C*_Q_ value. Although it is difficult to directly link aspects of the computationally generated structures to the calculated chemical shifts, the distributions of positions in Figure 4 suggests that the chemical shift trends could be influenced by the larger and more anisotropic range of displacements observed in the computationally generated oxygen positions. The clusters of oxygen positions imply that the dominant motion for these atoms is vibration nearly perpendicular to the P–O bond direction. Although the average bond lengths remain nearly constant in the neutron data, in the computational data this motion results in increasing P–O bond lengths with increasing temperature. For example, at 100 K, the distance from the computational positions was 1.5456 Å, and at 325 K, the average distance was 1.5527 Å (+0.0071 Å, +0.459%). This increase in the effective bond length is consistent with the increase in chemical shift observed in model 3 and the experimental NMR data. In the case of the Na–O bond lengths, at 100 K, the average distance was 2.4941 Å and at 325 K, the distance was 2.5134 Å (+0.0193 Å, +0.774%). Although these bond distances do not account for the fact that the sodium chemical shift increases with temperature, it does indicate one potential reason why the change in chemical shift in experiment is smaller than what would be expected from the bond distances in the neutron structures. Finally, while the values of *C*_Q_ are larger in the calculated results, the overall increase with temperature is nearly the same as in the experimental results. Therefore, while none of the NMR DFT calculations provide an exact match between theory and experiment, the model 3 calculations are the most consistent with experiment and demonstrate that the experimental trends in the NMR parameters between 100 and 300 K are mainly the result of changes in dynamics. One potential reason why the trends in the calculated NMR parameters are larger than what is observed experimentally is that model 3 relies on harmonic or self-consistent harmonic approximations. A more rigorous treatment of anharmonicity would lead to smaller changes in the NMR parameters, as model 3 likely overestimates the atomic displacements, as evidenced by the fact that the mean squared displacements are larger than the neutron *U* values for most sites. One way to accomplish this would be to run molecular dynamics (MD) simulations at each desired temperature and subsequently perform NMR calculations on each of the configurations generated. Unfortunately, this approach also has two disadvantages: first, MD does not account for quantum zero-point fluctuations unless path-integral MD is used, and second, the use of MD followed by NMR calculations would be extremely computationally expensive. As discussed above, we predict that the inclusion of anharmonicity would lead to improved agreement between experiment and computation, but the computational cost of the calculations would be too large for inclusion in this work.

## Conclusion

In this work we have used variable temperature neutron diffraction, NMR spectroscopy, and three sets of DFT calculations to improve our understanding of the thermal behavior of NaZr_2_(PO_4_)_3_. The structures determined from variable temperature neutron diffraction are similar to earlier models of thermal expansion in NASICON materials, where an increase in the Na–O bond distances with increasing temperature leads to an increase in the *c* lattice parameter, and rotations of rigid PO_4_ and ZrO_6_ polyhedra cause a decrease in the *a* lattice parameter. To understand the role of atomic vibrations in this model, we performed solid-state NMR experiments and attempted to use the atomic positions and thermal displacement ellipsoids calculated from the neutron data to interpret our results. Using empirically derived predictions for NMR parameters based on the neutron data was not successful in explaining the trends in the chemical shifts, so several DFT approaches were used instead. Model 1 matched the previously discussed empirical predictions based on the thermal expansion of the lattice but did not match the experimental trends. Similarly, model 2 showed slightly better results after relaxing atomic positions but did not allow us to identify the specific cause of the trends in the NMR spectra. Finally, the finite temperature calculations in model 3 produced values for the NMR parameters that matched all trends, at least qualitatively. Additionally, an analysis of the distribution of positions used in these calculations showed that they were consistent with the atomic displacement parameters obtained from the neutron refinements and provided some insight into the superior performance of model 3. In addition to providing a more complete model for the thermal expansion of NaZr_2_(PO_4_)_3_, these results illustrate several important concepts. The first is the sensitivity of solid-state NMR to changes in local dynamics with temperature. Second, these results suggest that first-principles phonon calculations can be useful in the prediction and interpretation of atomic displacement parameters from diffraction experiments. Finally, this work demonstrates the importance of properly accounting for finite temperature effects in DFT calculations of NMR parameters.

## Supporting Information Available

Selected bond lengths and angles from Rietveld refinements (Tables S1 and S2), complete variable-temperature NMR spectra and fits (Figures S1–S10), details of chemical shift calibrations (Figures S11–S14), comparisons of finite temperature structures and neutron refinements (Tables S3–S8), 2D projections of computationally generated structures (Figures S15 and S16), and summary of calculated NMR parameters from models 1–3 (Table S9, Figures S17 and S18) (PDF)
