# Supplementary material for: Lattice Dynamics in the NASICON NaZr2(PO4)3 Solid Electrolyte from Temperature-Dependent Neutron Diffraction, NMR, and Ab Initio Computational Studies
Source: Chem Mater. 2022 Apr 28;34(9):4029–38. doi: 10.1021/acs.chemmater.2c00212 (PMC9097157; doi:10.1021/acs.chemmater.2c00212)
Supplement: Supplementary file 1 — cm2c00212_si_001.pdf [file cm2c00212_si_001.pdf]

Supporting information for

Lattice Dynamics in the NASICON $NaZr_2(PO_4)_3$ Solid Electrolyte from Temperature-Dependent Neutron Diffraction, NMR, and Ab Initio Computational Studies

Emily E. Morgan,[1] Hayden A. Evans,[2] Kartik Pilar,[1] Craig M. Brown,[2] Raphaële J. Clément,[1] Ryo Maezono,[3] Ram Seshadri,[1,4] Bartomeu Monserrat ,[*,5,6] and Anthony K. Cheetham[*,1,7]

[1]Materials Department and Materials Research Laboratory, University of California, Santa Barbara, California 93106, United States

[2]NIST Center for Neutron Research, National Institute of Standards and Technology, Gaithersburg, Maryland 20878, United States

[3]School of Information Science, Japan Advanced Institute of Science and Technology, Asahidai 1-1, Nomi, Ishikawa 923-1292, Japan

[4]Department of Chemistry and Biochemistry

University of California, Santa Barbara, California 93106, United States

[5]Department of Materials Science and Metallurgy

University of Cambridge, 27 Charles Babbage Rd, Cambridge CB3 0FS, United Kingdom

[6]Cavendish Laboratory, University of Cambridge, J. J. Thomson Avenue, Cambridge CB3 0HE, United Kingdom

[7]Department of Materials Science and Engineering

National University of Singapore, Singapore 117575, Singapore

Table of contents

Section S1. Bond lengths and angles from Rietveld refinements

Section S2. Variable temperature NMR spectra and simulations

Section S3. Details for DFT chemical shift calibration curves

Section S4. Comparison of finite temperature calculated structures and neutron refinements

Section S5. 2D plots of finite temperature structures

Section S6. Summary of NMR calculation results

Section S1. Bond lengths and angles from Rietveld refinements

Temperature (K)	P1-O1 (Å)	P1-O2 (Å)	Zr1-O1 (Å)	Zr1-O2 (Å)	Na1-O2 (Å)
25	1.5272(19)	1.5322(16)	2.045(3)	2.0856(19)	2.5286(13)
100	1.5262(18)	1.5289(16)	2.040(2)	2.0937(18)	2.5345(13)
175	1.522(3)	1.536(2)	2.041(3)	2.092(3)	2.5381(16)
250	1.520(3)	1.530(2)	2.046(3)	2.091(3)	2.5467(16)
325	1.526(3)	1.525(3)	2.041(3)	2.085(3)	2.5643(18)
400	1.526(3)	1.522(3)	2.044(4)	2.083(3)	2.574(2)

Table S1 Selected bond lengths from 25 K to 400 K. Values in parentheses represent 1 standard deviation.

Temperature (K)	O1-P1-O1	O1-P1-O2	O2-P1-O2	O1-Zr1-O1	O1-Zr1-O2	O2-Zr1-O2
25	109.98(18)	110.34(8)	109.72(18)	92.19(10)	93.71(7)	83.46(9)
100	109.98(17)	108.13(9)	109.58(18)	92.43(9)	90.47(6)	83.31(9)
175	110.8(3)	110.57(9)	109.3(3)	92.60(11)	90.57(8)	83.21(10)
250	110.4(3)	107.90(12)	109.5(3)	92.30(12)	93.24(9)	83.50(11)
325	110.1(3)	108.30(13)	109.6(3)	92.12(13)	90.59(9)	84.10(12)
400	109.9(3)	108.02(14)	110.2(3)	91.82(13)	90.75(9)	84.32(12)

Table S2 Selected bond angles from 25 K to 400 K. Values in parentheses represent 1 standard deviation.

Section S2. Variable temperature NMR spectra and simulations

NMR spectra were simulated using the SOLA module in the Topspin† software package. For the ^{23}Na spectra, the Quad Central model was used to fit the spectra and determine the quadrupolar coupling constant and isotropic chemical shift at each temperature. In order to fully replicate the observed lineshape, two sites were required. The first site (1), which we attribute to the $NaZr_2(PO_3)_4$ is located around −14.2 ppm. The second site (2) is much lower in intensity and located at around 4-5 ppm in each spectrum. This second site is likely due to an amorphous impurity, as no secondary phase was observed in the neutron diffraction data. The ^{31}P spectra were fit using the CSA model. Two low-intensity peaks are visible at -21.9 ppm and -22.8 ppm, which we also attribute to the amorphous impurity phase.

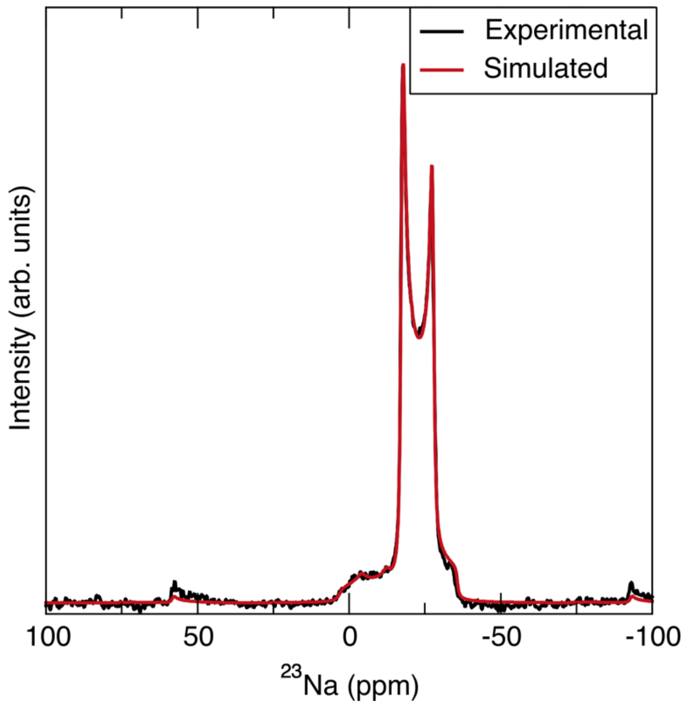

Figure S1 ^{23}Na spectrum at 100 K. (1) $\delta_{iso} = -14.23$ ppm, $C_Q = 1.958$ MHz, $\eta_Q = 0.067$. (2) $\delta_{iso} = 4.78$ ppm, $C_Q = 2.330$ MHz, $\eta_Q = 0.415$.

---

† Certain commercial equipment, instruments, or materials are identified in this document. Such identification does not imply recommendation or endorsement by the National Institute of Standards and Technology, nor does it imply that the products identified are necessarily the best available for the purpose.

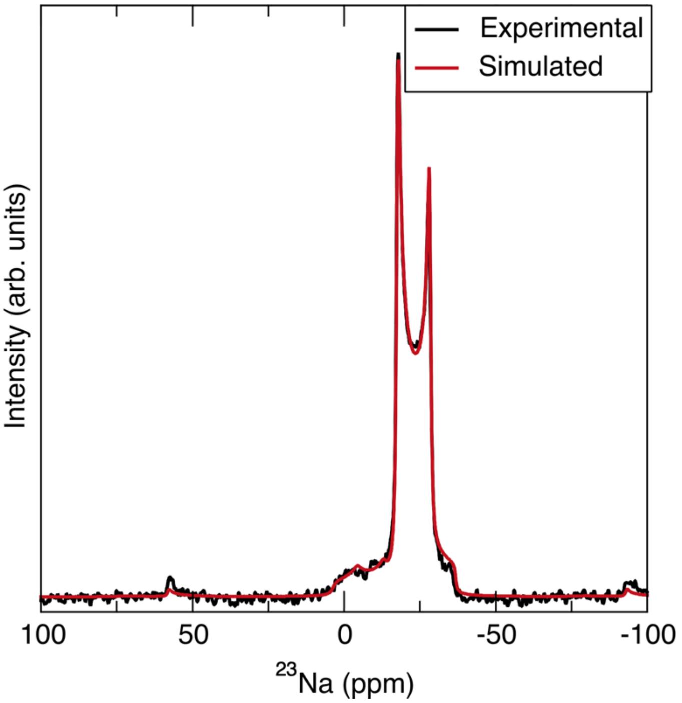

Figure S2 ^{23}Na spectrum at 140 K. (1) $\delta_{iso} =- 14.183$ ppm, $C_Q = 2.011$ MHz, $\eta_Q = 0.045$. (2) $\delta_{iso} = 5.258$ ppm, $C_Q = 2.395$ MHz, $\eta_Q = 0.44$.

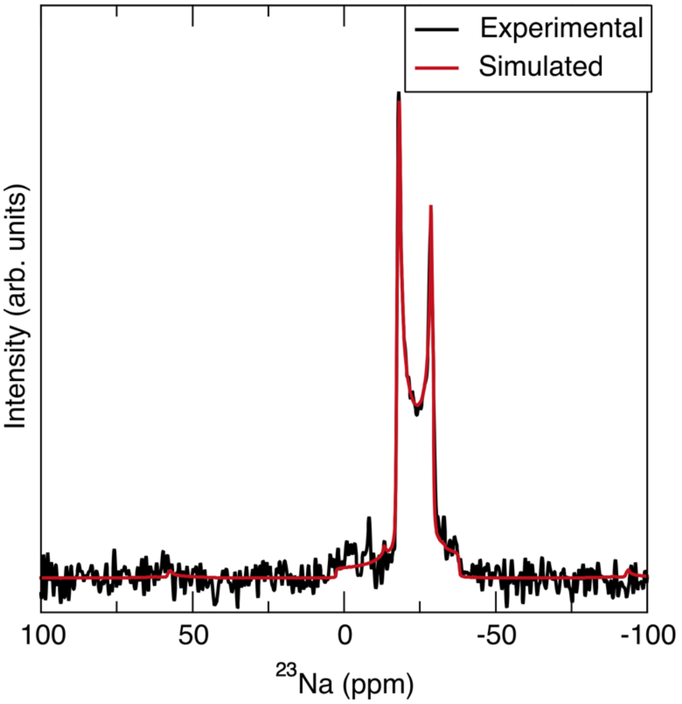

Figure S3 ^{23}Na spectrum at 179 K. (1) $\delta_{iso} =- 14.238$ ppm, $C_Q = 2.062$ MHz, $\eta_Q = 0.071$. (2) $\delta_{iso} = 3.109$ ppm, $C_Q = 2.586$ MHz, $\eta_Q = 0.75$.

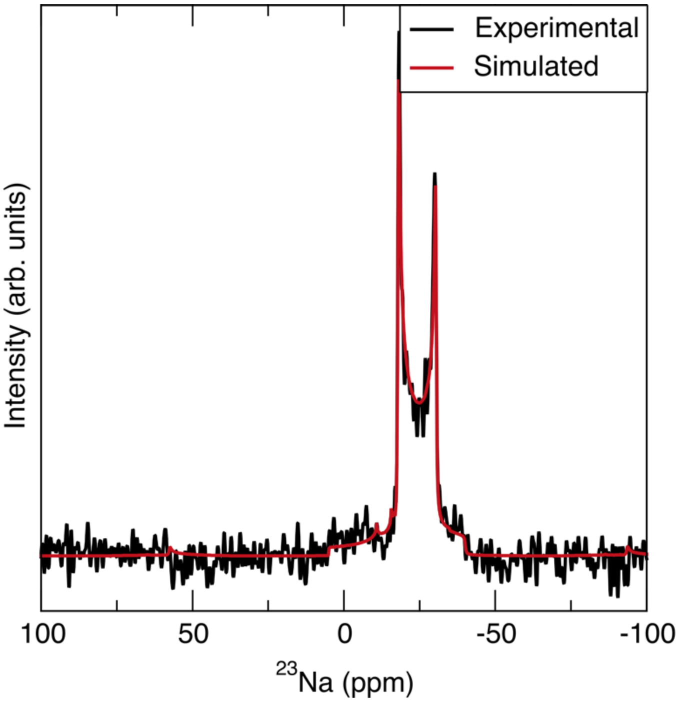

Figure S4 ^{23}Na spectrum at 232 K. (1) $\delta_{iso} =-14.165$ ppm, $C_Q = 2.154$ MHz, $\eta_Q = 0.0$. (2) $\delta_{iso} = 5.467$ ppm, $C_Q = 2.573$ MHz, $\eta_Q = 0.752$.

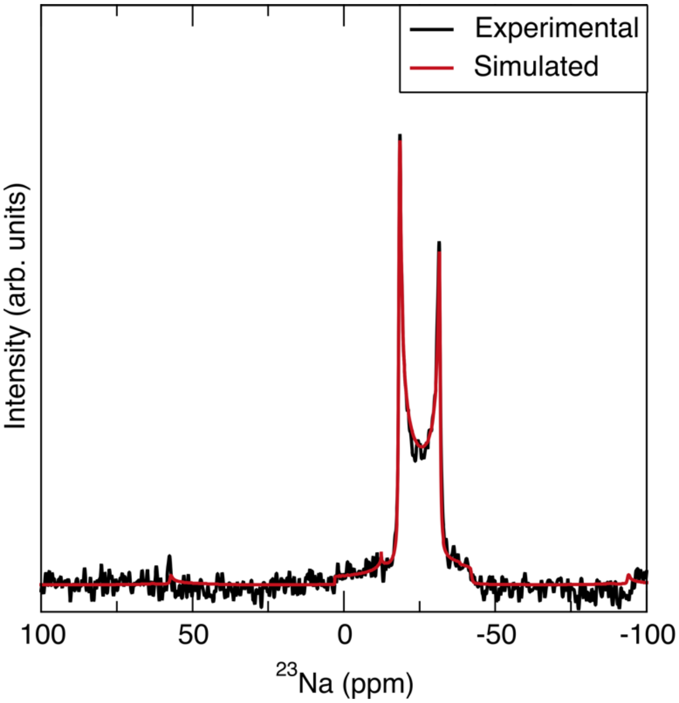

Figure S5 ^{23}Na spectrum at 314 K. (1) $\delta_{iso} =-14.178$ ppm, $C_Q = 2.241$ MHz, $\eta_Q = 0.022$. (2) $\delta_{iso} = 3.64$ ppm, $C_Q = 2.640$ MHz, $\eta_Q = 0.693$.

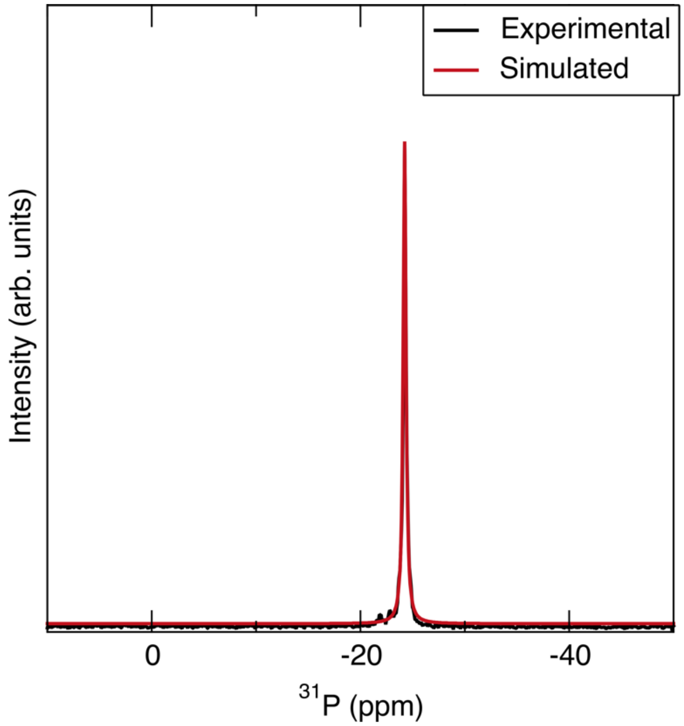

Figure S6 ^{31}P spectrum at 110 K. $\delta_{iso} = -24.19$ ppm

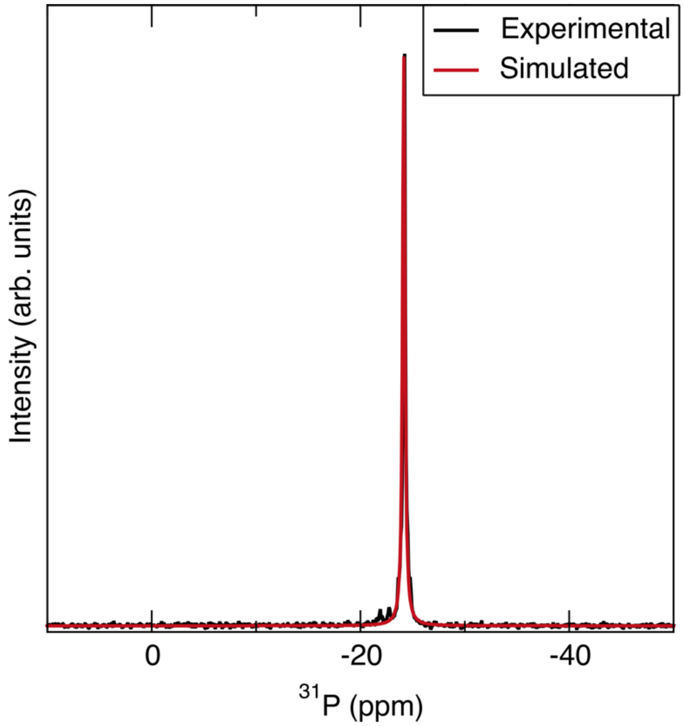

Figure S7 ^{31}P spectrum at 137 K. $\delta_{iso} = -24.16$ ppm

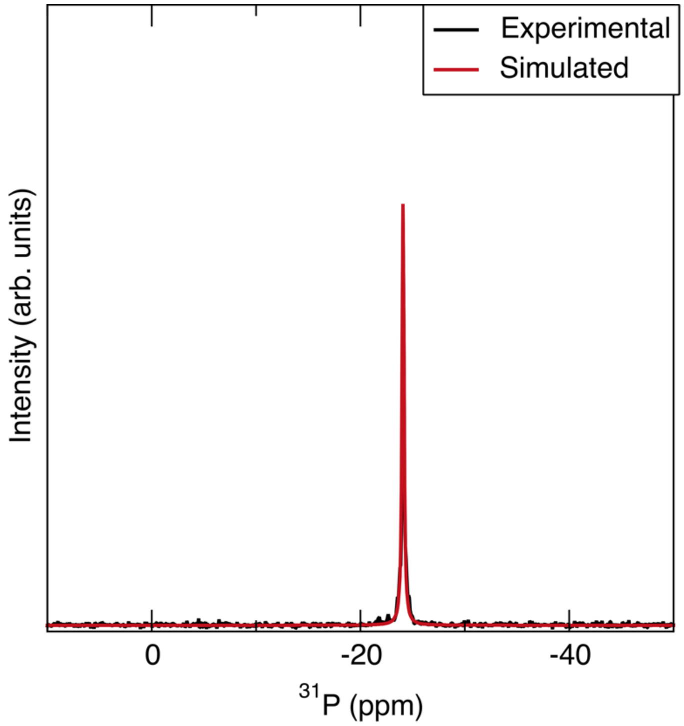

Figure S8 ^{31}P spectrum at 187 K. $\delta_{iso} = -24.08$ ppm

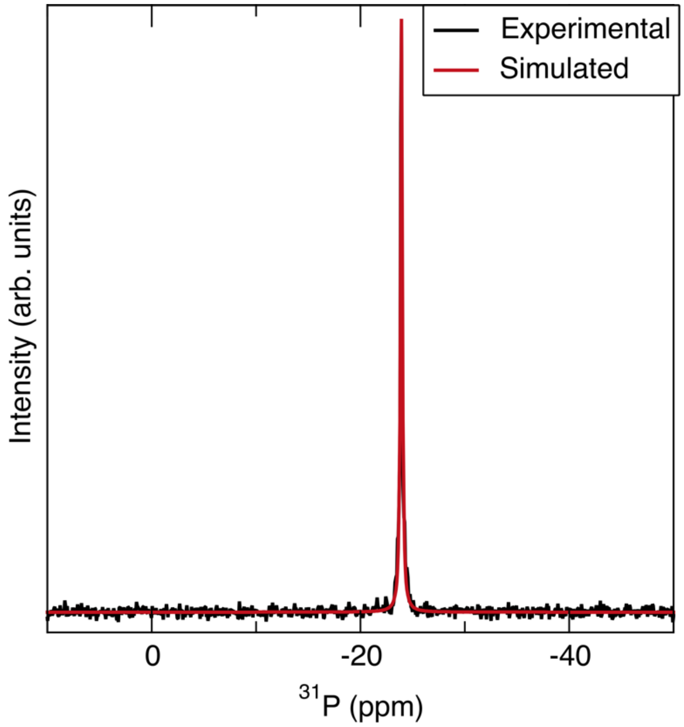

Figure S9 ^{31}P spectrum at 232 K. $\delta_{iso} = -23.92$ ppm

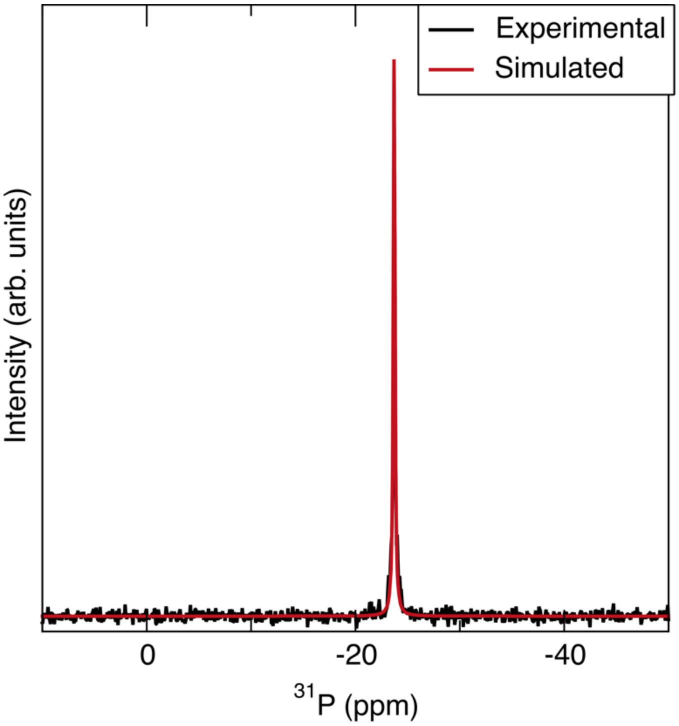

Figure S10 ^{31}P spectrum at 312 K. $\delta_{iso} = -23.66$ ppm

Section S3. Chemical shift calibration curves for Model 1 and Model 2

In order to construct the chemical shift calibration curves for Models 1 and 2, crystal structures were obtained from the Inorganic Crystal Structure Database[1–31] and the relevant experimental chemical shift values were taken from the literature.[32–38] For Model 1, structures were downloaded and converted to the .cell file format using the CIF2Cell python package.[39] For Model 2, the same structures were first relaxed in the Vienna Ab initio Simulation Package[40–42] using the recommended PAW potentials[43,44], an energy cutoff of 600 eV, and a k-point grid with length parameter of 40.[45] The lattice parameters were held fixed at the experimentally-determined values and the internal coordinates of the atoms were relaxed until all forces were smaller than 10^{-4} eV/Å. Following relaxation, each structure was converted to the .cell file format in order to perform the NMR calculations. Chemical shifts for all models were calculated using density functional theory[46,47] and the PBE functional[48] as implemented in the CASTEP package,[49] using an energy cutoff of 1000 eV, a k-point grid spacing of 2π*0.025 Å,[45] and ultrasoft pseudopotentials.[50]

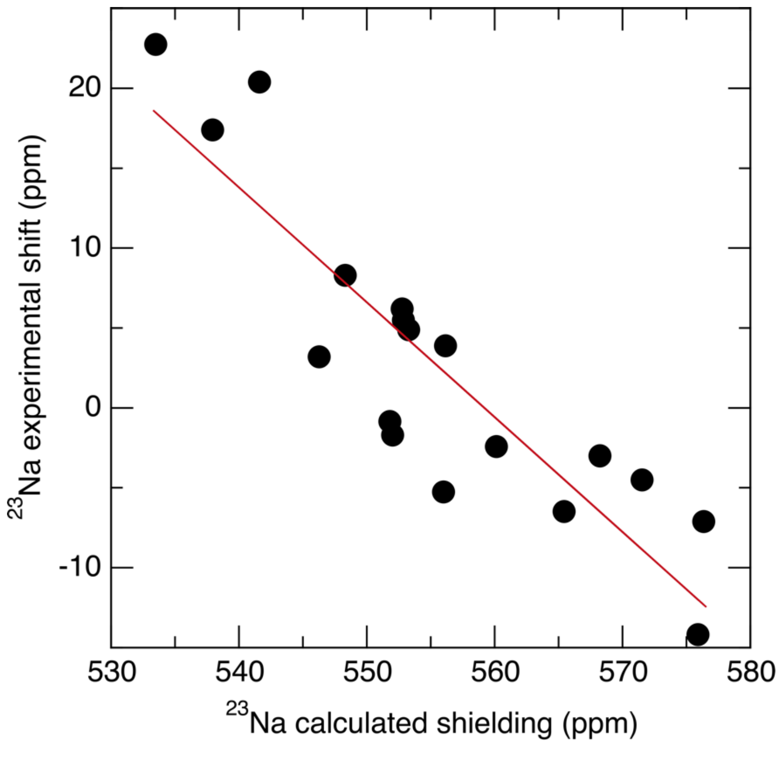

Figure S11 ^{23}Na chemical shift calibration curve for unrelaxed structures (Model 1). The regression line has a slope of -0.71842, an intercept of 401.74 ppm, and a correlation coefficient of -0.8919.

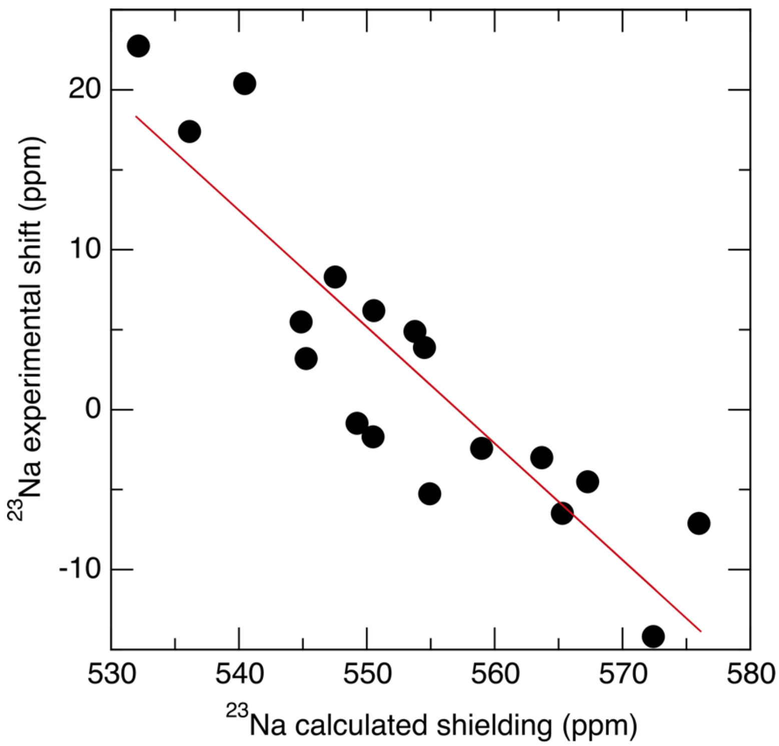

Figure S12 ^{23}Na chemical shift calibration curve for relaxed structures (Model 2). The regression line has a slope of -0.72852, an intercept of 405.86 ppm, and a correlation coefficient of -0.8891.

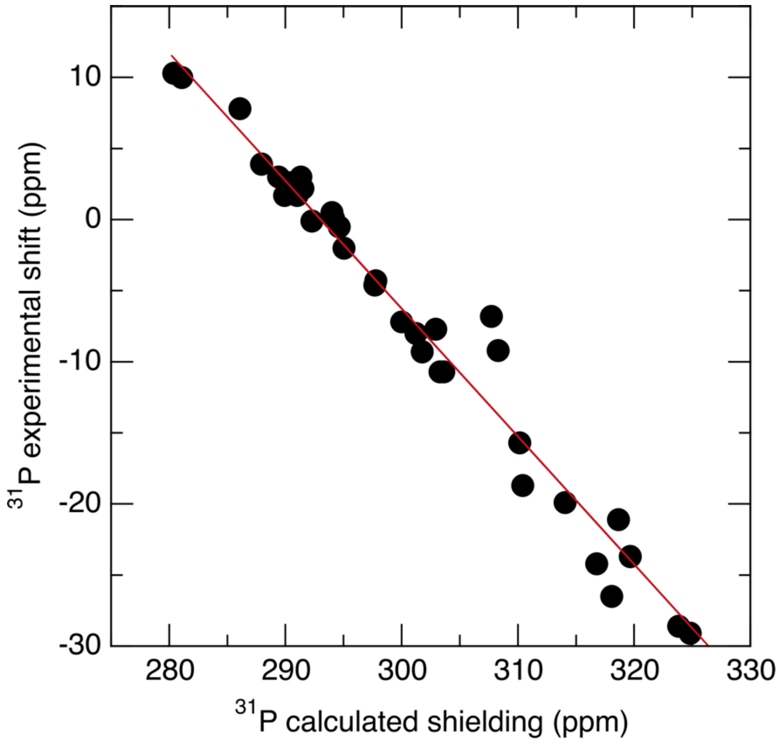

Figure S13 ^{31}P chemical shift calibration curve for unrelaxed structures (Model 1). The regression line has a slope of -0.89933, an intercept of 263.54 ppm, and a correlation coefficient of -0.9885475.

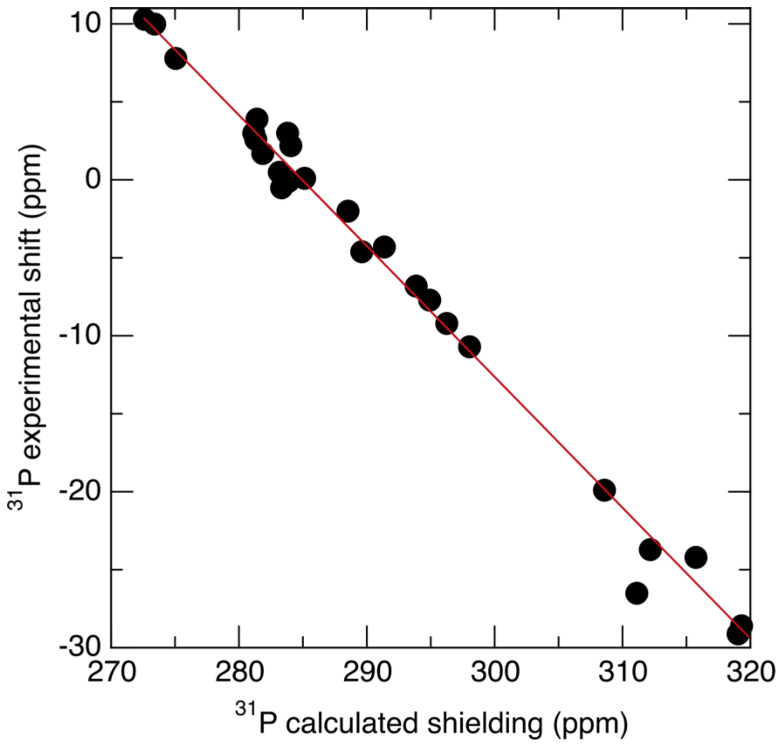

Figure S14 ^{31}P chemical shift calibration curve for relaxed structures (Model 2). The regression line has a slope of -0.83908, an intercept of 239.09 ppm, and a correlation coefficient of -0.9954.

Section S4. Comparison of finite temperature structures and neutron refinements

Temperature (K)	$\langle a,b^2 \rangle$ displacement (Å^2)	$\langle c^2 \rangle$ displacement (Å^2)	Average mean square displacement (Å^2)	$U_{iso(neutron)}$ (Å^2)
25	0.0535	0.0541	0.0537	0.0057, 0.00575
100	0.0779	0.0747	0.0766	0.006, 0.00598
175	0.116	0.132	0.122	0.0072, 0.0059
250	0.155	0.174	0.161	0.0102, 0.0072
325	0.197	0.218	0.204	0.0135, 0.0105
400	0.238	0.267	0.248	0.0161, 0.0134

Table S3 Comparison of mean squared displacements calculated for O with experimental isotropic atomic displacement parameters from neutron refinements.

Temperature (K)	$\langle a,b^2 \rangle$ (Å^2)	$\langle c^2 \rangle$ (Å^2)	U_{11} (neutron) (Å^2)	U_{22} (neutron) (Å^2)	U_{33} (neutron) (Å^2)	U_{12} (neutron) (Å^2)	U_{13} (neutron) (Å^2)	U_{23} (neutron) (Å^2)
325	0.197	0.218	0.0122, 0.0056	0.0177, 0.0092	0.0129, 0.0122	0.0091, 0.0003	-0.0071, 0.0025	0.0031, - 0.0003
400	0.238	0.267	0.0177 0.0073	0.0206, 0.0104	0.0125, 0.0159	0.0114, -0.0006	-0.0086, 0.0029	0.0038, 0.0017

Table S4 Comparison of mean squared displacements calculated for O with experimental anisotropic atomic displacement parameters from neutron refinements.

Temperature (K)	$<a,b^2>$ displacement (Å^2)	$<c^2>$ displacement (Å^2)	$<a,b^2>/<c^2>$	U_{11} (neutron) (Å^2)	U_{33} (neutron) (Å^2)	U_{11}/U_{33}
25	0.00747	0.00363	2.06	0.0085	0.0085	1.0
100	0.0121	0.00450	2.69	0.0164	0.0071	2.31
175	0.0195	0.00618	3.16	0.0234	0.0110	2.13
250	0.0273	0.00818	3.34	0.0333	0.0040	8.33
325	0.0362	0.0106	3.42	0.0600	0.0150	4.00
400	0.0453	0.0125	3.62	0.0740	0.0140	5.28

Table S5 Comparison of mean squared displacements calculated for Na with experimental atomic displacement parameters from neutron refinements.

Temperature (K)	$<a,b^2>$ displacement (Å^2)	$<c^2>$ displacement (Å^2)	Average mean square displacement (Å^2)	$U_{iso(neutron)}$ (Å^2)
25	0.00498	0.00655	0.00547	0.0039
100	0.00664	0.00870	0.00730	0.0042
175	0.00990	0.0189	0.0129	0.0032
250	0.0126	0.0245	0.0166	0.0047
325	0.0155	0.0302	0.0204	0.0031
400	0.0188	0.0377	0.0251	0.0048

Table S6 Comparison of mean squared displacements calculated for P with experimental atomic displacement parameters from neutron refinements.

Temperature (K)	$<a,b^2>$ displacement (Å^2)	$<c^2>$ displacement (Å^2)	Average mean square displacement (Å^2)	$U_{iso(neutron)}$ (Å^2)
25	0.00292	0.00350	0.00311	0.00582
100	0.00455	0.00433	0.00446	0.00458
175	0.00760	0.0122	0.00908	0.0032
250	0.0101	0.0157	0.0119	0.003
325	0.0125	0.0198	0.0148	0.00506
400	0.0148	0.0246	0.0182	0.00548

Table S7 Comparison of mean squared displacements calculated for Zr with experimental atomic displacement parameters from neutron refinements.

Temperature (K)	<a,b^2> (Å^2)	<c^2> (Å^2)	U_{11} (neutron) (Å^2)	U_{22} (neutron) (Å^2)	U_{33} (neutron) (Å^2)	U_{12} (neutron) (Å^2)	U_{13} (neutron) (Å^2)	U_{23} (neutron) (Å^2)
325	0.0125	0.0198	0.0038	0.0038	0.0076	0.00192	0	0
400	0.0148	0.0246	0.0054	0.0054	0.0056	0.0027	0	0

Table S8 Comparison of mean squared displacements calculated for Zr with experimental anisotropic atomic displacement parameters from neutron refinements.

Section S5. 2D plots of finite temperature structures

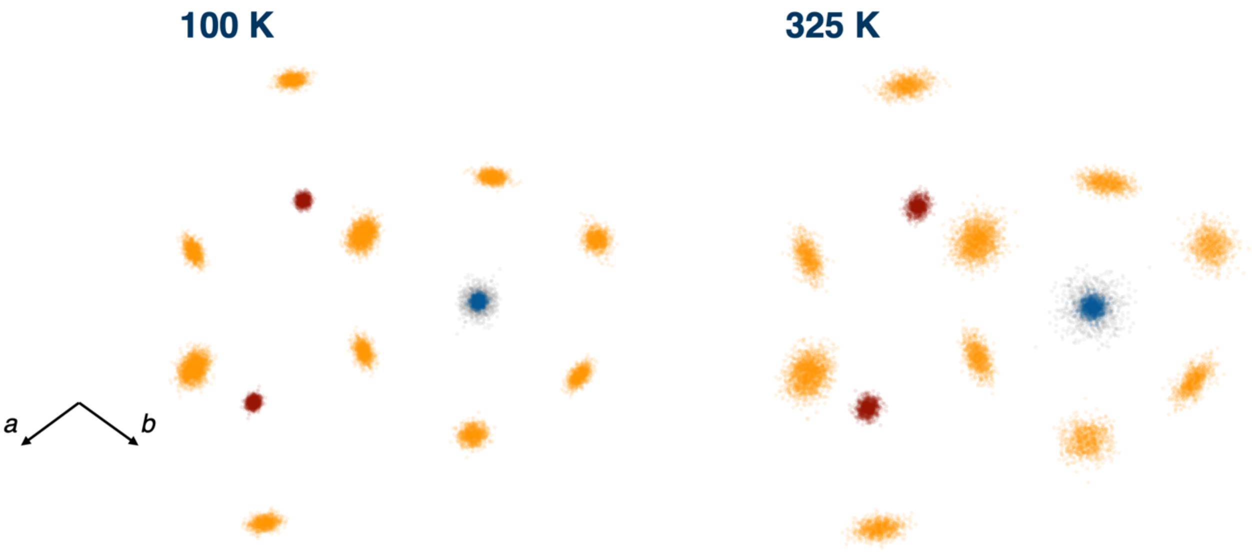

Figure S15 2D distribution of computationally-generated atomic positions projected onto the *a*-*b* plane (looking down the *c* axis) at 100 K and 325 K.

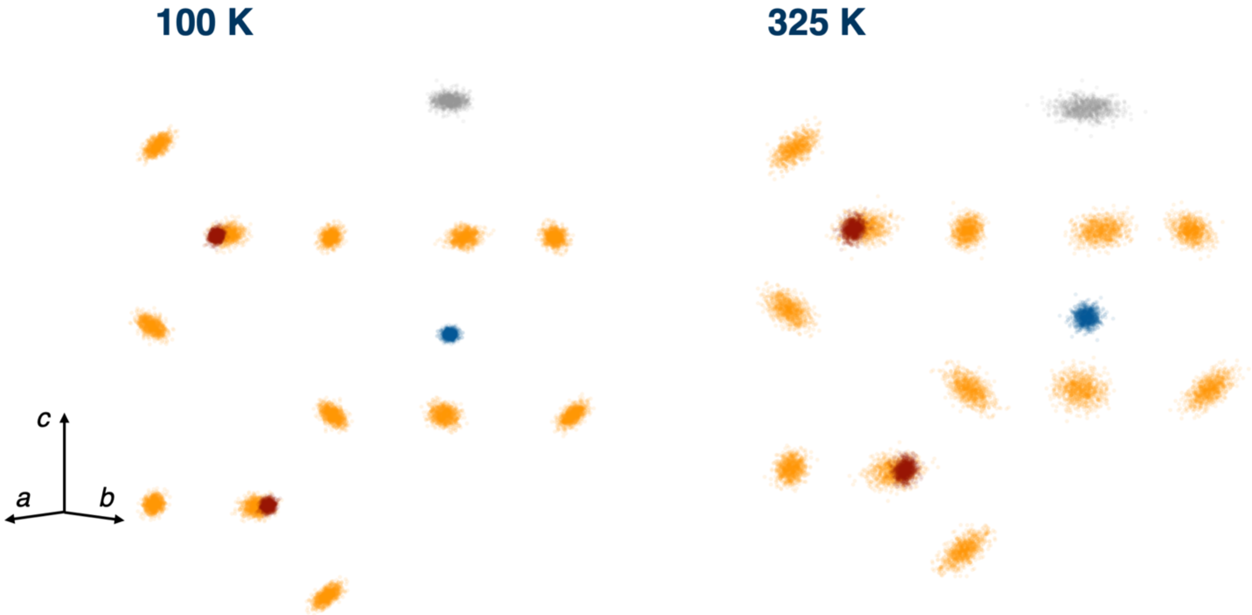

Figure S16 2D distribution of computationally-generated atomic positions projected onto the *c* axis (looking along the axis which bisects the *a* and *b* axes) at 100 K and 325 K.

Section S6. Summary of NMR calculation results

Parameter	Temperature (K)	Model 1	Model 2	Model 3
^{23}Na chemical shift (ppm)	25	−11.14	−10.34	−14.57
	100	−11.51	−10.38	−14.30
	175	−12.30	−10.56	−13.75
	250	−12.39	−10.90	−13.46
	325	−13.59	−11.25	−13.13
	400	−14.61	−11.66	
^{23}Na C_Q (MHz)	25	2.11	2.17	2.24
	100	2.10	2.18	2.25
	175	2.17	2.20	2.39
	250	2.11	2.21	2.44
	325	2.13	2.23	2.56
	400	2.12	2.25	
^{31}P chemical shift (ppm)	25	−25.02	−25.14	−25.51
	100	−25.46	−25.13	−24.21
	175	−25.05	−25.11	−23.78
	250	−26.18	−25.06	−22.47
	325	−25.39	−25.02	−21.03
	400	−25.56	−24.97	

Table S9 Summary of calculated NMR parameters (as displayed in Figure 7 of the main text). Chemical shift values for Models 1 and 2 are re-scaled and shifted according to the calibration curves shown in Figures S11-S14. The chemical shift values for Model 3 are shifted by a constant amount such that the experimental and calculated values match at 100 K.

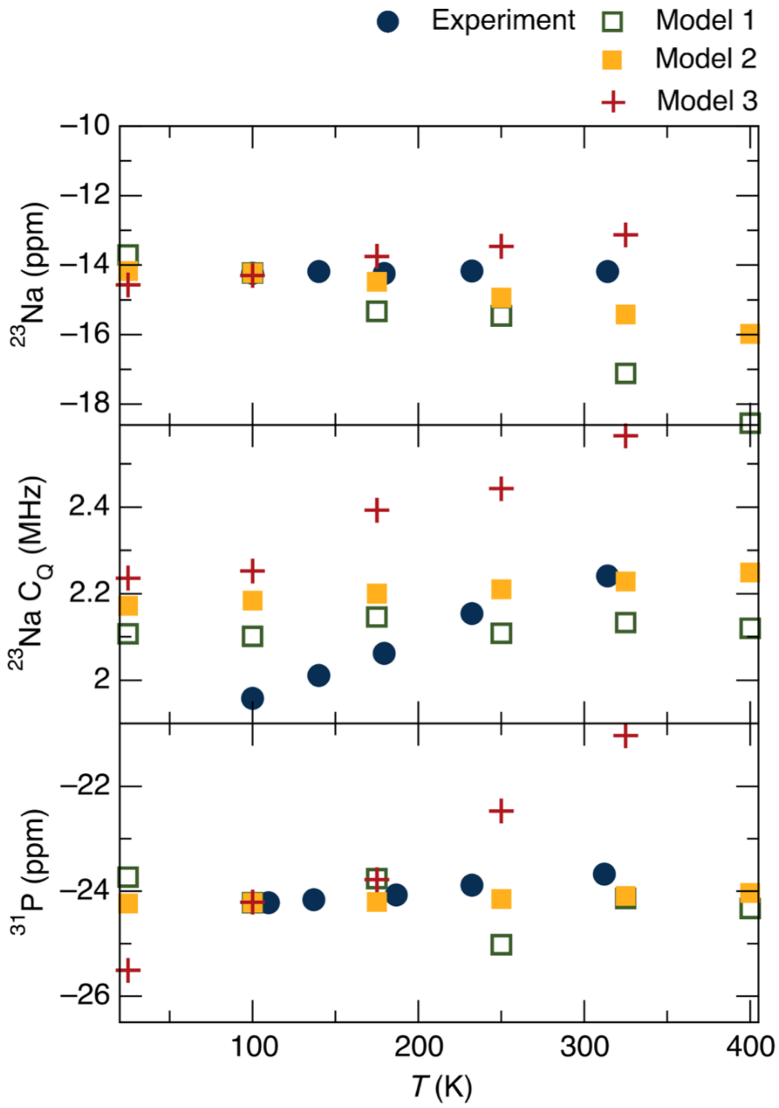

Figure S17 Comparison of calculated and experimental chemical shifts and C_Q values. Here, rather than shifting and rescaling Models 1 and 2 according to the calibration curves, the chemical shift values are shifted by a constant amount to match the experimental 100 K values to provide a clearer comparison with Model 3. Values for Model 3 and all C_Q values are the same as Figure 7.

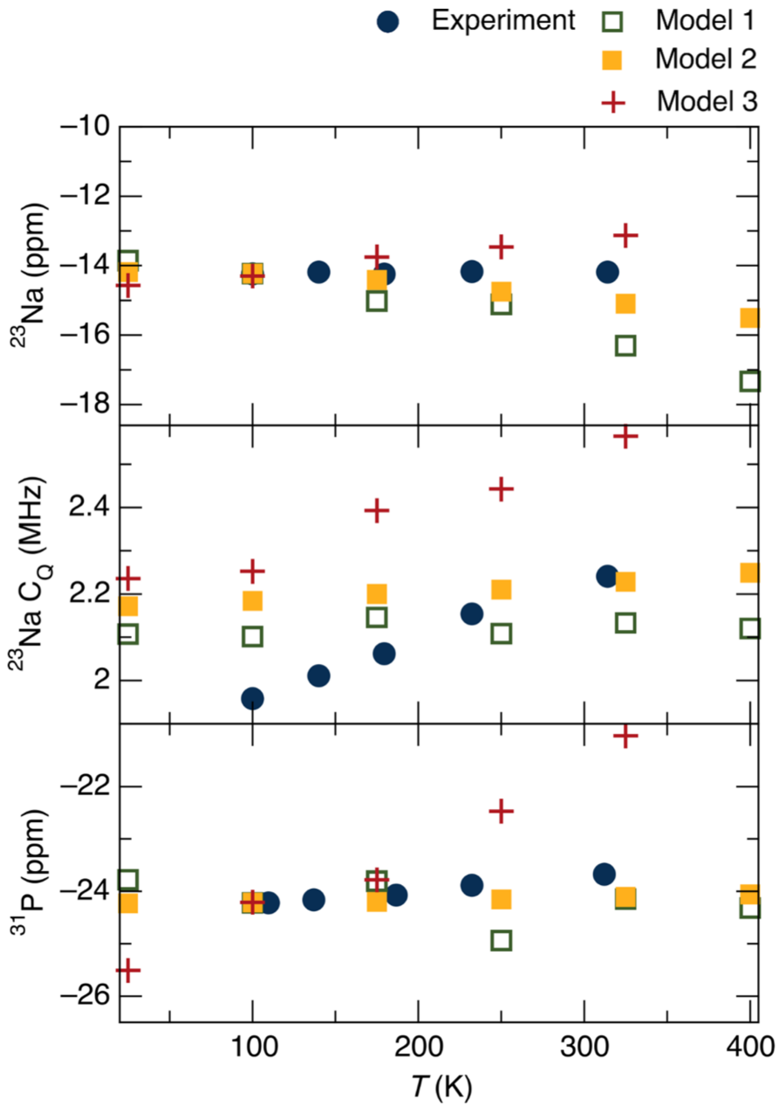

Figure S18 Comparison of calculated and experimental chemical shifts and C_Q values. Here, the chemical shift values for Models 1 and 2 are re-scaled by the values determined from the calibration curves and then shifted to match the experimental values at 100 K. Values for Model 3 and all C_Q values are the same as Figure 7.

(1) Ayu, N. I. P.; Kartini, E.; Prayogi, L. D.; Faisal, M.; Supardi. Crystal Structure Analysis of Li_3PO_4 Powder Prepared by Wet Chemical Reaction and Solid-State Reaction by Using X-Ray Diffraction (XRD). *Ionics* **2016**, *22* (7), 1051–1057.
(2) Calvo, C.; Au, P. K. L. Crystal Structure of $Cd_2P_2O_7$. *Can. J. Chem.* **1969**, *47* (18), 3409–3416.
(3) Dickens, B.; Prince, E.; Schroeder, L. W.; Brown, W. E. $Ca(H_2PO_4)_2$, a Crystal Structure Containing Unusual Hydrogen Bonding. *Acta Cryst B* **1973**, *29* (10), 2057–2070.
(4) Calvo, C. The Crystal Structure of α-$Zn_3(PO_4)_2$. *Can. J. Chem.* **1965**, *43* (2), 436–445.
(5) Leung, K. Y.; Calvo, C. The Structure of $Na_4P_2O_7$ at 22 °C. *Can. J. Chem.* **1972**, *50* (16), 2519–2526.
(6) Jakeman, R. J. B.; Cheetham, A. K. Combined Single-Crystal x-Ray Diffraction and Magic Angle Spinning NMR Study of α -$CaZn_2(PO_4)_2$. *J. Am. Chem. Soc.* **1988**, *110* (4), 1140–1143.
(7) Averbuch-Pouchot, M. T. Structure Du Monophosphate Acide de Potassium–Zinc: $KZn_2H(PO_4)_2$. *Acta Cryst B* **1979**, *35* (6), 1452–1454.
(8) Stephens, J. S.; Calvo, C. Crystal Structure of β-$Zn_3(PO_4)_2$. *Can. J. Chem.* **1967**, *45* (20), 2303–2316.
(9) Schroeder, L. W.; Prince, E.; Dickens, B. Hydrogen Bonding in $Ca(H_2PO_4)_2$•H_2O as Determined by Neutron Diffraction. *Acta Cryst B* **1975**, *31* (1), 9–12.
(10) A. Curry, N.; W. Jones, D. Crystal Structure of Brushite, Calcium Hydrogen Orthophosphate Dihydrate: A Neutron-Diffraction Investigation. *Journal of the Chemical Society A: Inorganic, Physical, Theoretical* **1971**, *0* (0), 3725–3729.
(11) Nord, A. G. The Cation Distribution in $Zn_2Mg(PO_4)_2$ Determined by X-Ray Profile-Fitting Refinements. *Materials Research Bulletin* **1977**, *12* (6), 563–568.
(12) Boudin, S.; Grandin, A.; Borel, M. M.; Leclaire, A.; Raveau, B. Redetermination of the β-$Ca_2P_2O_7$ Structure. *Acta Cryst C* **1993**, *49* (12), 2062–2064.
(13) Calvo, C. Crystal Structure of α -Calcium Pyrophosphate. *Inorg. Chem.* **1968**, *7* (7), 1345–1351.
(14) Nord, A. G.; Kierkegaard, P. The Crystal Structure of $Mg_3(PO_4)_3$. *Acta Chem. Scand. A* **1968**, *22*, 1466–1474.
(15) Robertson, B. E.; Calvo, C. Crystal Structure of $AZn_2P_2O_7$. *Journal of Solid State Chemistry* **1970**, *1* (2), 120–133.
(16) Calvo, C. The Crystal Structure of α-$Mg_2P_2O_7$. *Acta Cryst* **1967**, *23* (2), 289–295.
(17) Alkemper, J.; Paulus, H.; Fueβ, H. Crystal Structure of Aluminum Sodium Pyrophosphate, $NaAlP_2O_7$. *Zeitschrift für Kristallographie - Crystalline Materials* **1994**, *209* (7), 616.
(18) Ng, H. N.; Calvo, C. The Crystal Structure of $KAlP_2O_7$. *Can. J. Chem.* **1973**, *51* (16), 2613–2620.
(19) Hagman, L. O.; Kierkegaard, P. The Crystal Structure of $NaMe_2(PO_4)_3$; Me-Ge, Ti, Zn. *Acta Chem. Scand.* **1968**, *22*, 1822–1832.
(20) Hagman, L. O.; Jansson, I.; Magnéli, C. The Crystal Structure of α-$Sr_2P_2O_7$. *Acta Chem. Scand.* **1968**, *22*, 1419–1429.
(21) Nord, A. G.; Lindberg, K. B. The Crystal Structure of Magnesium Tetrametaphosphate, $Mg_2P_4O_{12}$. *Acta Chem. Scand. A* **1975**, *29*, 1–6.
(22) Onac, B. P.; Effenberger, H. S. Re-Examination of Berlinite ($AlPO_4$) from the Cioclovina Cave, Romania. *American Mineralogist* **2007**, *92* (11–12), 1998–2001.
(23) He, M.; Chen, X. L.; Zhou, T.; Hu, B. Q.; Xu, Y. P.; Xu, T. Crystal Structure and Infrared Spectra of $Na_2Al_2B_2O_7$. *Journal of Alloys and Compounds* **2001**, *327* (1), 210–214.
(24) McDonald, W. S.; Cruickshank, D. W. J. A Reinvestigation of the Structure of Sodium Metasilicate, Na_2SiO_3. *Acta Cryst* **1967**, *22* (1), 37–43.
(25) Pant, A. K.; Cruickshank, D. W. J. The Crystal Structure of α-$Na_2Si_2O_5$. *Acta Cryst B* **1968**, *24* (1), 13–19.

(26) Pant, A. K. A Reconsideration of the Crystal Structure of β-$Na_2Si_2O_5$. *Acta Cryst B* **1968**, *24* (8), 1077–1083.
(27) Catti, M.; Ferraris, G. Hydrogen Bonding in the Crystalline State. NaH_2PO_4, a Crystal Structure with a Short Symmetrical Hydrogen Bond. *Acta Cryst B* **1974**, *30* (1), 1–6.
(28) Baldus, M.; Meier, B. H.; Ernst, R. R.; Kentgens, A. P. M.; Meyer zu Altenschildesche, H.; Nesper, R. Structure Investigation on Anhydrous Disodium Hydrogen Phosphate Using Solid-State NMR and X-Ray Techniques. *J. Am. Chem. Soc.* **1995**, *117* (18), 5141–5147.
(29) Mitchell, R. H.; Burns, P. C.; Knight, K. S.; Howard, C. J.; Chakhmouradian, A. R. Observations on the Crystal Structures of Lueshite. *Phys Chem Minerals* **2014**, *41* (6), 393–401.
(30) Mitchell, R. H.; Liferovich, R. P. A Structural Study of the Perovskite Series $Ca_{1-x}Na_xTi_{1-x}Ta_xO_3$. *Journal of Solid State Chemistry* **2004**, *177* (12), 4420–4427.
(31) Selevich, K. A.; Ivashkevich, L. S.; Selevich, A. F.; Lyakhov, A. S. Refinement of Crystal Structure of $Na_2H_2P_2O_7$ Using X-Ray Powder Diffraction Data. *Zhurnal Neorganicheskoi Khimii* **2002**, *47* (10), 1672–1675.
(32) Cheetham, A. K.; Clayden, N. J.; Dobson, C. M.; Jakeman, R., J. B. Correlations between ^{31}P N.M.R. Chemical Shifts and Structural Parameters in Crystalline Inorganic Phosphates. *J. Chem. Soc., Chem. Comm.* **1986**, No. 3, 195–197.
(33) Pilar, K.; Deng, Z.; Preefer, M. B.; Cooley, J. A.; Clément, R.; Seshadri, R.; Cheetham, A. K. *Ab Initio* Computation for Solid-State ^{31}P NMR of Inorganic Phosphates: Revisiting X-Ray Structures. *Phys. Chem. Chem. Phys.* **2019**, *21* (19), 10070–10074.
(34) Perras, F. A.; Bryce, D. L. Multinuclear Magnetic Resonance Crystallographic Structure Refinement and Cross-Validation Using Experimental and Computed Electric Field Gradients: Application to $Na_2Al_2B_2O_7$. *J. Phys. Chem. C* **2012**, *116* (36), 19472–19482.
(35) Charpentier, T.; Ispas, S.; Profeta, M.; Mauri, F.; Pickard, C. J. First-Principles Calculation of ^{17} O, ^{29} Si, and ^{23} Na NMR Spectra of Sodium Silicate Crystals and Glasses. *J. Phys. Chem. B* **2004**, *108* (13), 4147–4161.
(36) Engelhardt, G.; Kentgens, A. P. M.; Koller, H.; Samoson, A. Strategies for Extracting NMR Parameters from ^{23}Na MAS, DOR and MQMAS Spectra. A Case Study for $Na_4P_2O_7$. *Solid State Nuclear Magnetic Resonance* **1999**, *15* (3), 171–180.
(37) Vasconcelos, F.; Cristol, S.; Paul, J.-F.; Montagne, L.; Mauri, F.; Delevoye, L. First-Principles Calculations of NMR Parameters for Phosphate Materials. *Magn. Reson. Chem.* **2010**, *48* (S1), S142–S150.
(38) E. Ashbrook, S.; Pollès, L. L.; Gautier, R.; J. Pickard, C.; I. Walton, R. ^{23}Na Multiple-Quantum MAS NMR of the Perovskites $NaNbO_3$ and $NaTaO_3$. *Physical Chemistry Chemical Physics* **2006**, *8* (29), 3423–3431.
(39) Björkman, T. CIF2Cell: Generating Geometries for Electronic Structure Programs. *Computer Physics Communications* **2011**, *182* (5), 1183–1186.
(40) Kresse, G.; Hafner, J. *Ab Initio* Molecular-Dynamics Simulation of the Liquid-Metal–Amorphous-Semiconductor Transition in Germanium. *Phys. Rev. B* **1994**, *49* (20), 14251–14269.
(41) Kresse, G.; Furthmüller, J. Efficiency of Ab-Initio Total Energy Calculations for Metals and Semiconductors Using a Plane-Wave Basis Set. *Computational Materials Science* **1996**, *6* (1), 15–50.
(42) Kresse, G.; Furthmüller, J. Efficient Iterative Schemes for *Ab Initio* Total-Energy Calculations Using a Plane-Wave Basis Set. *Phys. Rev. B* **1996**, *54* (16), 11169–11186.
(43) Kresse, G.; Joubert, D. From Ultrasoft Pseudopotentials to the Projector Augmented-Wave Method. *Phys. Rev. B* **1999**, *59* (3), 1758–1775.

(44) Blöchl, P. E. Projector Augmented-Wave Method. *Phys. Rev. B* **1994**, *50* (24), 17953–17979.
(45) Monkhorst, H. J.; Pack, J. D. Special Points for Brillouin-Zone Integrations. *Phys. Rev. B* **1976**, *13* (12), 5188–5192.
(46) Hohenberg, P.; Kohn, W. Inhomogeneous Electron Gas. *Phys. Rev.* **1964**, *136* (3B), B864–B871.
(47) Kohn, W.; Sham, L. J. Self-Consistent Equations Including Exchange and Correlation Effects. *Phys. Rev.* **1965**, *140* (4A), A1133–A1138.
(48) Perdew, J. P.; Burke, K.; Ernzerhof, M. Generalized Gradient Approximation Made Simple. *Phys. Rev. Lett.* **1996**, *77* (18), 3865–3868.
(49) Clark, S. J.; Segall, M. D.; Pickard, C. J.; Hasnip, P. J.; Probert, M. I. J.; Refson, K.; Payne, M. C. First Principles Methods Using CASTEP. *Zeitschrift für Kristallographie - Crystalline Materials* **2005**, *220* (5/6).
(50) Vanderbilt, D. Soft Self-Consistent Pseudopotentials in a Generalized Eigenvalue Formalism. *Phys. Rev. B* **1990**, *41* (11), 7892–7895.
